# *ACE2* and *TMPRSS2* SARS-CoV-2 infectivity genes: deep mutational scanning and characterization of missense variants

**DOI:** 10.1093/hmg/ddac157

**Published:** 2022-07-21

**Authors:** Lingxin Zhang, Vivekananda Sarangi, Duan Liu, Ming-Fen Ho, Angela R Grassi, Lixuan Wei, Irene Moon, Robert A Vierkant, Nicholas B Larson, Konstantinos N Lazaridis, Arjun P Athreya, Liewei Wang, Richard Weinshilboum

**Affiliations:** Division of Clinical Pharmacology, Department of Molecular Pharmacology and Experimental Therapeutics, Mayo Clinic, Rochester, MN 55905, USA; Division of Clinical Trials and Biostatistics, Department of Quantitative Health Sciences, Mayo Clinic, Rochester, MN 55905, USA; Division of Clinical Pharmacology, Department of Molecular Pharmacology and Experimental Therapeutics, Mayo Clinic, Rochester, MN 55905, USA; Division of Clinical Pharmacology, Department of Molecular Pharmacology and Experimental Therapeutics, Mayo Clinic, Rochester, MN 55905, USA; Department of Medicine, Mayo Clinic, Rochester, MN 55905, USA; Division of Clinical Pharmacology, Department of Molecular Pharmacology and Experimental Therapeutics, Mayo Clinic, Rochester, MN 55905, USA; Division of Clinical Pharmacology, Department of Molecular Pharmacology and Experimental Therapeutics, Mayo Clinic, Rochester, MN 55905, USA; Division of Clinical Trials and Biostatistics, Department of Quantitative Health Sciences, Mayo Clinic, Rochester, MN 55905, USA; Division of Clinical Trials and Biostatistics, Department of Quantitative Health Sciences, Mayo Clinic, Rochester, MN 55905, USA; Center for Individualized Medicine, Mayo Clinic, Rochester, MN 55905, USA; Division of Gastroenterology and Hepatology, Department of Medicine, Mayo Clinic, Rochester, MN 55905, USA; Division of Clinical Pharmacology, Department of Molecular Pharmacology and Experimental Therapeutics, Mayo Clinic, Rochester, MN 55905, USA; Center for Individualized Medicine, Mayo Clinic, Rochester, MN 55905, USA; Division of Clinical Pharmacology, Department of Molecular Pharmacology and Experimental Therapeutics, Mayo Clinic, Rochester, MN 55905, USA; Department of Medicine, Mayo Clinic, Rochester, MN 55905, USA; Division of Clinical Pharmacology, Department of Molecular Pharmacology and Experimental Therapeutics, Mayo Clinic, Rochester, MN 55905, USA; Center for Individualized Medicine, Mayo Clinic, Rochester, MN 55905, USA

## Abstract

The human angiotensin-converting enzyme 2 (ACE2) and transmembrane serine protease 2 (TMPRSS2) proteins play key roles in the cellular internalization of severe acute respiratory syndrome coronavirus 2 (SARS-CoV-2), the coronavirus responsible for the coronavirus disease of 2019 (COVID-19) pandemic. We set out to functionally characterize the ACE2 and TMPRSS2 protein abundance for variant alleles encoding these proteins that contained non-synonymous single-nucleotide polymorphisms (nsSNPs) in their open reading frames (ORFs). Specifically, a high-throughput assay, deep mutational scanning (DMS), was employed to test the functional implications of nsSNPs, which are variants of uncertain significance in these two genes. Specifically, we used a ‘landing pad’ system designed to quantify the protein expression for 433 nsSNPs that have been observed in the *ACE2* and *TMPRSS2* ORFs and found that 8 of 127 *ACE2*, 19 of 157 *TMPRSS2* isoform 1 and 13 of 149 *TMPRSS2* isoform 2 variant proteins displayed less than ~25% of the wild-type protein expression, whereas 4 *ACE2* variants displayed 25% or greater increases in protein expression. As a result, we concluded that nsSNPs in genes encoding ACE2 and TMPRSS2 might potentially influence SARS-CoV-2 infectivity. These results can now be applied to DNA sequence data for patients infected with SARS-CoV-2 to determine the possible impact of patient-based DNA sequence variation on the clinical course of SARS-CoV-2 infection.

## Introduction

The coronavirus disease of 2019 (COVID-19) pandemic had resulted in >6 221 000 deaths worldwide by April 2022. Severe acute respiratory syndrome coronavirus 2 (SARS-CoV-2), the virus responsible for this disease, infects human cells after recognition and internalization facilitated by human angiotensin-converting enzyme 2 (ACE2), a protein encoded by the *ACE2* gene (1). During that process, the SARS-CoV-2 virus is primed by transmembrane serine protease 2 (*TMPRSS2*) to facilitate virus entry (2,3). Genetic polymorphisms of *ACE2* and *TMPRSS2*, which affect the expression of these two genes have been associated with COVID-19 clinical outcomes (4–6). For example, over-expression of the *ACE1* receptor as well as *ACE2* down regulation by intronic variant rs2285666, results in *ACE1*/*ACE2* imbalance, contributing to pulmonary failure (7,8). In addition, a recent genome-wide association study (GWAS) reported that the *ACE2* upstream intronic SNP (rs190509934) downregulated ACE2 expression by 37% (*P* = 2.7 × 10^−8^) and reduced the risk of SARS-CoV-2 infection by 40% (9). A common *TMPRSS2* nsSNP, rs12329760 (minor allele frequency = 0.25 across different populations), has been associated with protection against severe COVID-19 symptoms (10,11). As a result, it will be important to study genetic variation in the *ACE2* and *TMPRSS2* genes because of the possibility of their impact on the susceptibility to SARS-CoV-2 internalization. Those genetic variations in both genes could alter the disease course for COVID-19 patients (12,13), and could be one of the factors contributing to individual variation in viral infectivity.

Hundreds of non-synonymous single-nucleotide polymorphisms (nsSNPs) in the *ACE2* and *TMPRSS2* genes have been reported by the gnomAD database (14). Those nsSNPs result in amino acid substitutions in the encoded protein. However, the majority of the nsSNPs that have been observed in the *ACE2* and *TMPRSS2* are ‘variants of uncertain significance’. To address this challenge, predictive algorithms such as Polyphen-2, SIFT and PROVEAN have been applied as well as structural modeling strategies to help identify variants of interest in terms of their effect on protein expression or function (15–17). However, laboratory-based assays are necessary to provide the objective evidence required to reliably interpret the impact of nsSNPs in these genes on protein function. A common deleterious effect of nsSNPs on the encoded protein is altered protein structure and resultant rapid degradation (18,19). For decades, a standard method to study the effect of nsSNPs on protein level has been to clone the nsSNP cDNA plasmid and to overexpress the protein isoform in a cellular environment to compare its protein level with that of the WT protein. However, that approach is time-consuming and labor-intensive and, as a result, cannot practically be applied to study the hundreds of nsSNPs that have been reported in the *ACE2* and *TMPRSS2* genes.

In order to avoid time-consuming and labor-intensive ‘one-at-a-time’ characterization of nsSNP effect on the encoded protein, next-generation DNA sequencing (NGS)-based deep mutational scanning (DMS) assays have been developed to characterize the functional implications of nsSNPs at scale (20,21). DMS makes it possible to pool and assay multiple nsSNPs in parallel to examine their protein expression levels using a cell-based ‘landing pad’ system. DMS involves the creation of nsSNP cDNA over-expression libraries for the gene being studied, fluorescence-activated cell sorting (FACS) based on abundance of reporter protein expression, and finally the use of high-throughput NGS to read out the nsSNPs and link them to protein abundance scores as a test of function (20,21). Using this approach, we have previously successfully functionally characterized hundreds of nsSNPs in three important ‘pharmacogenes’, *CYP2C9*, *CYP2C19* and *SLCO1B1* (22,23).

In the present study, we functionally characterized 127 nsSNPs in *ACE2*, 157 in *TMPRSS2* isoform 1 and 149 in *TMPRSS2* isoform 2, nsSNPs, which were reported in the publicly available gnomAD browser (https://gnomad. broadinstitute.org/) that included exome sequence data for 70 000 subjects at the time that we designed this study (24). We found that the *TMPRSS2* rs12329760 nsSNP resulted in a significant decrease in its encoded protein level, which might help to explain why the rs12329760 SNP has been associated with decreased disease severity for COVID-19 (10,11). In addition, we found that 8 of the *ACE2* nsSNPs resulted in ~75% decreases, whereas 4 *ACE2* nsSNPs displayed 25% or greater increases in protein expression levels when compared with the wild-type protein. We also found that 19 of the *TMPRSS2* isoform 1 and 13 *TMPRSS2* isoform 2 variant proteins displayed <25% of the wild-type protein level. These results might help to determine the possible impact of patient-based DNA sequence variation for these two genes on the clinical course of SARS-CoV-2 infection.

## Results

### Generation of *ACE2* and *TMPRSS2* variant libraries

**Figure 1:**
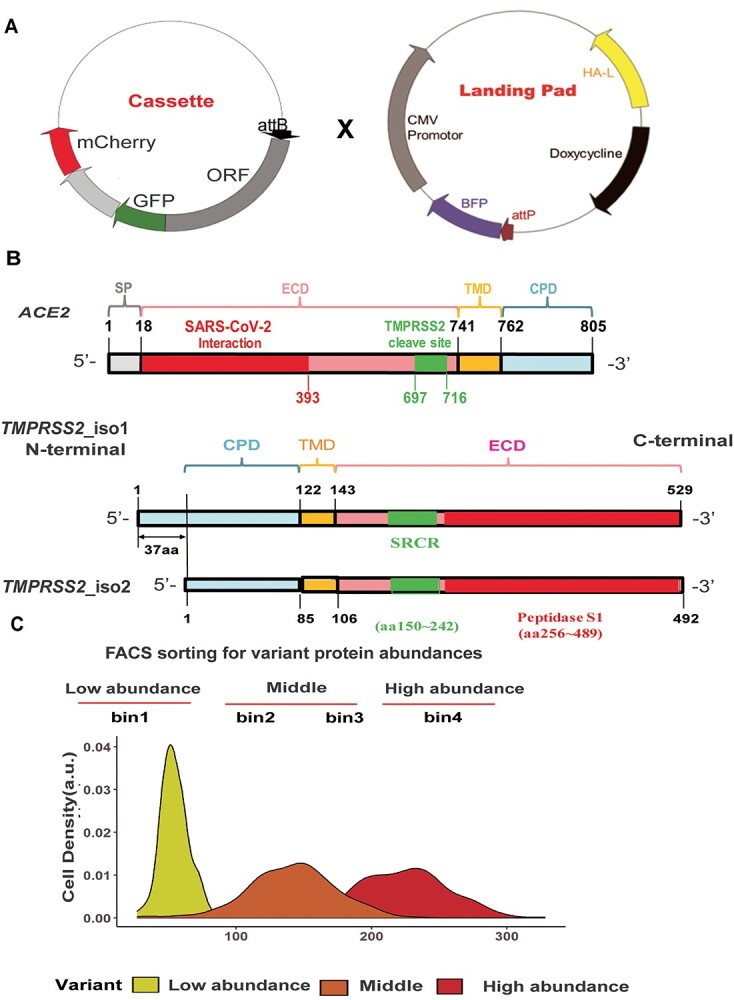
DMS of variant libraries for *ACE2* and *TMPRSS2*. (**A**) Plasmid maps of the landing pad construct and the promoter-less cassette for ORFs that were fused to GFP and engineered for the simultaneous expression of IRES-mCherry are shown. (**B**) Gene structures for *ACE2* and *TMPRSS2* (isoforms 1 and 2) are shown schematically. SP = signal peptide, CPD = cytoplasmic domain, ECD = extracellular domain, TMD = transmembrane domain and SRCR = scavenger receptor cysteine-rich domain. (**C**) Flow cytometry analysis of BFP^−^/mCherry^+^ cells integrated with pooled variant libraries. FACS sorting BFP^−^/mCherry^+^ cells into 4 bins based on their GFP/mCherry ratios. Gates were set based on WT *ACE2* or *TMPRSS2*. Pools of sorted cells in each bin were collected and were used as input material for subsequent amplicon DNA sequencing. High-abundance variants eluted toward higher GFP/mCherry ratios in bin 4, whereas variants in bin 1 contained low-abundance variants that eluted at significantly lower GFP/mCherry ratios than did cells containing the WT.

HEK293T cell clone #20 with a genome that had integrated our ‘landing pad’ platform was generated previously [(22,23); see Fig. 1A]. That cell line contained one copy of the ‘landing pad’ per cell, enabling the ‘landing’ of one promoter-less expression cassette per cell by DNA recombination. The successful ‘landing’ of a promoter-less cassette would replace the BFP expression sequence. The promoter-less cassette containing the open reading frames (ORF) of *ACE2* and the C-terminal ORF was fused to GFP as were the ORFs of the two *TMPRSS2* isoforms (isoforms 1 and 2), followed by mCherry as an internal control. After transfection into cell line clone #20, the promoter-less cassette was only able to express ACE2 or TMPRSS2 proteins when it was integrated into the ‘landing pad’, which contained a CMV promoter and, in the process, disrupted BFP expression (see Fig. 1A). Specifically, the pooled nsSNPs containing *ACE2* or *TMPRSS2* promoter-less cassette constructs were transfected into ‘landing pad’ clone #20, followed by single cell sorting based on the expression of fluorescent reporter protein (BFP^−^/mCherry^+^). The gene structures for *ACE2* or *TMPRSS2* (isoforms 1 and 2) are shown schematically in Figure 1B. TMPRSS2 isoform 2 is 37 amino acids shorter at the N-terminus than is isoform 1 as a result of alternative splicing of the mRNA encoding isoform 2. Both of the TMPRSS2 isoforms are expressed in lung, heart, liver and the gastrointestinal tract, etc. (25,26). Some of the variants studied expressed reduced levels of GFP and lower GFP/mCherry ratios, indicating that those cells expressed less protein than did cells transfected with WT (see Fig. 1C). We created constructs for nsSNPs with minor allele frequencies (MAF) >  0.00001 as reported by the gnomAD browser (versions 2.0 and 3.0) for both *ACE2* and *TMPRSS2*. Pooled variant libraries for *ACE2* or isoforms 1 and 2 for *TMPRSS2* were integrated into landing pad cells, BFP^−^/mCherry^+^ cells, and were collected as pool variant libraries (see Supplementary Material, Table S3). To evaluate protein abundance using the WT *ACE2* or *TMPRSS2* constructs as references for FACS gating, the BFP^−^/mCherry^+^ cells were sorted into four ‘bins’ based on GFP/mCherry ratios, bins related to ACE2 or TMPRSS2 protein expression. Variants of *ACE2*/*TMPRSS2* with the lowest GFP/mCherry ratios (<25% protein expression) were sorted into bin 1 and were classified as low-abundance variants. WT-like variants were sorted into bin 4. The gating for 4-way sorting of *ACE2*/*TMPRSS2* pooled variant libraries for protein expression is shown graphically in Figure 1C.

DNA was collected for the cells in each bin and was used as input material for NGS amplicon sequencing to calculate variant frequencies (*F_v_*) in each bin. Abundance scores for each *ACE2* and *TMPRSS2* variant were calculated by use of the *F_v_* for variants by multiplying the variant frequency by weighted values from 0.25 to 1, with 0.25 assigned to bin 1 and with bin 4 assigned a value of 1, as indicated in the following equation:

Abundance score = $\frac{\big({F}_{v, bin1}\times 0.25\big)+\big({F}_{v, bin2}\times 0.5\big)+\big({F}_{v, bin3}\times 0.75\big)+\big({F}_{v, bin4}\times 1\big)}{\big({F}_{v, bin1}+{F}_{v, bin2}+{F}_{v, bin3}+{F}_{v, bin4.}\big)}$

**Figure 2:**
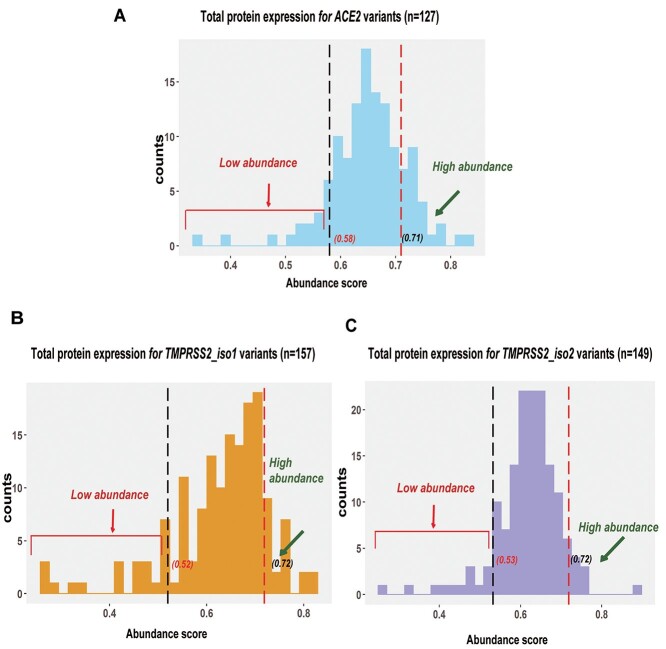
Protein abundance scores for 127 *ACE2*, 157 *TMPRSS2* isoform 1 and 149 *TMPRSS2* isoform 2 variants. (**A**) Abundance score values for *ACE2* variant protein expression. Variants having the top 20 high or low-abundance scores were used for further validation. The results shown are averages of at least three replicates. (**B**) Abundance score values for *TMPRSS2* isoform 1 variant protein expression. (**C**) Abundance score values for *TMPRSS2* isoform 2 variant protein expression. The results shown are averages of at least three replicates. SD values are listed in Supplementary Material, Tables S4–S6.

The final abundance score for each variant was calculated by averaging mean abundance scores across at least three replicate assays. The protein abundance scores for *ACE2* or *TMPRSS2* (isoforms 1 and 2) were determined by the use of massively parallel sequencing for all variants in gnomAD (v2.0 and v3.0) with MAF >  0.00001 and are listed in Supplementary Material, Tables S1 and S2. The abundance scores for *ACE2/TMPRSS2* (isoforms 1 and 2) variants are shown graphically in Figure 2. Abundance scores and confidence intervals for *ACE2/TMPRSS2* (isoforms 1 and 2) variants from four replicates are listed in Supplementary Material, Tables S4–S6.

### Validation of low or high-abundance variants for *ACE2* or *TMPRSS2*

**Figure 3:**
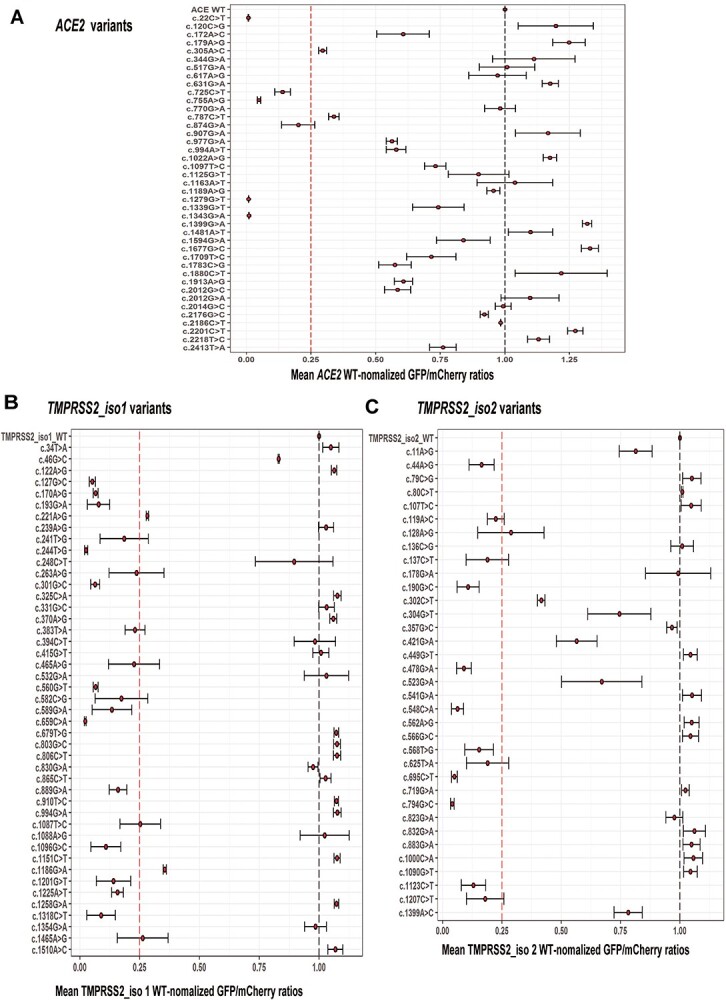
Flow cytometry validation of *ACE2* and *TMPRSS2* variants with the top 20 high-or low-abundance scores for protein expression (**A**) Mean GFP/mCherry ratios of BFP^−^/mCherry^+^ cells expressing *ACE2* variants with high or low-abundance scores were validated individually by flow cytometry. The mean GFP/mCherry ratios were normalized to the WT Mean GFP/mCherry ratio, indicating the protein expression. (**B**) Mean GFP/mCherry ratios of BFP^−^/mCherry^+^ cells expressing *TMPRSS2* isoform 1 variants with high or low-abundance scores were validated individually by flow cytometry. The mean GFP/mCherry ratios of variants were normalized to the WT Mean GFP/mCherry ratio, indicating the level of protein expression. (**C**) Mean GFP/mCherry ratios of BFP^−^/mCherry^+^ cells expressing *TMPRSS2* isoform 2 variants with high or low-abundance scores were validated individually by flow cytometry. The mean GFP/mCherry ratios of variants were normalized to the WT Mean GFP/mCherry ratio, indicating the level of protein expression.

The efficiency of massively parallel sequencing significantly exceeds the throughput of traditional mutagenesis methods. However, we still wanted to confirm the accuracy of calling for the variants that we studied. Therefore, the functional impact of variants associated with the top 20 high or low protein abundance scores for *ACE2* (low cutoff: 0.58, high cutoff: 0.71), *TMPRSS2* isoform 1 (low cutoff: 0.52, high cutoff: 0.72) and *TMPRSS2* isoform 2 (low cutoff: 0.53, high cutoff: 0.72) variants were validated by flow cytometry. Mean GFP/mCherry ratios for the top 20 high or low protein abundance constructs for *ACE2* or *TMPRSS2* (isoforms 1 and 2) variants were compared with those of the WT proteins, as shown graphically in Figure 3 A–C for each variant. In Figure 3, we have highlighted variants with lower than or higher than 25% of the mean WT GFP/mCherry ratios, percentages that are potentially of clinical significance, as we have reported previously (22,23). The variants of interest for both genes are shown at the far left or far right in each of the panels. We found that 8 of 127 *ACE2*, 19 of 157 *TMPRSS2* isoform 1 and 13 of 149 *TMPRSS2* isoform 2 variants displayed less than ~25% of the WT protein expression, whereas 4 *ACE2* variants displayed a 25% or greater elevation of protein expression.

**Figure 4:**
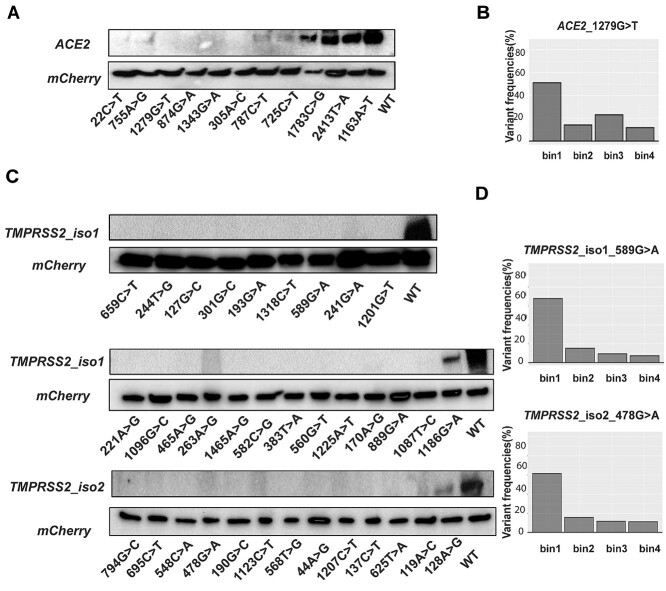
Western blot validation of *ACE2* and *TMPRSS2* constructs identified as containing low protein abundance variants. (**A**) The protein expression of *ACE2* in BFP^−^/mCherry^+^ cells integrating low-abundance variants was validated by western blot analysis. *ACE2* (2413 T > A and 1163A > T) are variants with middle abundance. Each image in this figure includes a control lane for wild-type (WT) protein assayed by western blot analysis. (**B**) The panel shows *F_v_* by bin for one representative low-abundance *ACE2* variant (rs1316056737, 1279G > T). (**C**) The protein expression of *TMPRSS2* (isoforms 1 and 2) in BFP^−^/mCherry^+^ cells integrating low-abundance variants were validated by western blot analysis. mCherry was used as a loading control. Each image includes a control lane for wild-type (WT). (**D**) Variant frequency distributions of *TMPRSS2* rs12329760 isoform 1 (589G > A) and isoform 2 (478G > A) into each of the four bins.

Our DMS results were also compared with *in silico* prediction results obtained using predictions of the SIFT, Provean, Polyphen2 and CADD algorithms and those results are listed in Supplementary Material, Table S1 for *ACE2* and in Supplementary Material, Table S2 for *TMPRSS2*, respectively. For variants that resulted in dramatically reduced protein expression levels, *ACE2* rs200745906 and another 5 variants for *ACE2*; 7 of 19 *TMPRSS2* variants for isoform 1 and only 1 of 13 variants for *TMPRSS2* isoform 2 were in agreement across all three predictive algorithms. As shown in Figure 4A and C, our newly identified low-abundance variants for *ACE2* and *TMPRSS2* displayed significantly decreased protein expression (near or less than 25%) compared with the WT protein. The binning patterns for *F_v_* for selected variants, *ACE2* (rs1316056737, 1279G > T) and *TMPRSS2* rs12329760 isoform 1 (589G > A) and *TMPRSS2* isoform 2 (478G > A) are shown in Figure 4B and D. The rs1016777825 (1677G > C) variant for human *ACE2* displayed 25% increased protein expression (Fig. 3A). This variant maps to the binding site for the SARS-CoV-2 spike protein, a portion of the protein related to host-viral interaction and the sensing of viral RNAs (27). The rs1316056737 variant resulted in < 25% ACE2 protein expression and mapped to a portion of the gene encoding the SARS-CoV-2 receptor-binding domain (28,29). Darbani *et al.* have also identified rs1316056737 as an interaction inhibitor variant that might impact the interaction between the *ACE2* and the SARS-CoV-2 viral spike protein (30). DMS results for TMPRSS2 (isoforms 1 and 2) suggested that the *TMPRSS2* rs12329760 SNP resulted in < 25% protein expression. Notably, a GWAS meta-analysis from the COVID-19 Host Genetics Initiative (31), a study that combined genetic and clinical phenotype data from 49 562 cases and 2 million controls across 46 studies from 19 countries reported that subjects carrying the *TMPRSS2* rs12329760 homozygous variant genotype had a lower susceptibility for COVID and a lower probability of developing severe respiratory symptoms compared with subjects homozygous for the wild-type genotype as confirmed by COVID cases versus a population control (log(OR) = −0.10, *P* = 8.18 × 10^−6^); hospitalized COVID cases versus population controls (log(OR) = −0.06, *P* = 4.72 × 10^−6^); and hospitalized COVID versus non-hospitalized COVID (log(OR) = −0.04, *P* = 0.012). Recent structural modelling of *TMPRSS2* rs12329760 also showed that the protein which it encodes displays decreased *TMPRSS2* stability and there is also additional evidence showing lower SARS-CoV-2 infection rates in an Indian population for this variant (11). A study from UK intensive care units as part of the GenOMICC (Genetics of Mortality In Critical Care) study reported an association between the rs12329760 variant and protective effects in COVID-19 clinical severity (10). Those data are consistent with our DMS results for TMPRSS2 (isoforms 1 and 2) and suggest that *TMPRSS2* rs12329760 may decrease both COVID susceptibility and severity. As a result, our study provides additional information with regard to the functional implications of these variants, information which might help make it possible to predict the infectivity and clinical outcome of SNVs that have not previously been reported or which have uncertain functional implications.

## Discussion

The role of sequence variation in the genes encoding human *ACE2* and *TMPRSS2* in susceptibility to SARS-CoV-2 infection has not been comprehensively examined experimentally and remains a challenge in the current pandemic. In a recent series of studies, we identified and functionally characterized *CYP2C9*, *CYP2C19* and *SLCO1B1* missense variants using a DMS platform. We found that the enzyme activities of the *CYP2C9* and *CYP2C19* variants generally correlated well with protein expression levels. We hypothesized that SNVs might also affect the abundance of ACE2 and *TMPRSS2 protein expression* and, as a result, change the susceptibility of individuals to SARS-CoV2 infection (32,33). Therefore, we applied our established DMS platform, which makes it possible to study the functional implications of a large number of missense variants by analyzing abundance of the encoded protein by fluorescence in a parallel manner through the use of FACS and NGS (see Fig. 1). Specifically, we analyzed and generated abundance scores for 433 human genome non-synonymous ORF variants for *ACE2* and *TMPRSS2* obtained from the gnomAD study (V2.0 and V3.0) that had MAF values > 0.00001, frequencies that are not so rare as to be ‘private’ (see Supplementary Material, Tables S1 and S2).

Our previous publications and those of others (21–23,34) have demonstrated that DMS appears to be useful for identifying low-abundance variants for protein such as *CYP2C9* and *CYP2C19*, which are subject to rapid protein degradation, often proteasome-mediated (18,19), and which can display clear fluorescence separation from protein encoded by the WT sequence. A limitation of DMS based on fluorescence is that some genes, which encode transmembrane proteins, such as *SLCO1B1*, *ACE2* and *TMPRSS2* require careful interpretation and the validation of variants that display expression level changes. The validation of functional studies for variants characterized in this fashion will be essential if we are to incorporate these results into clinical decision-making and electronic health records. To validate the low-abundance or high-abundance variants that we identified by DMS, we used western blot assays and/or flow cytometry to validate variants of interest, even though those studies were laborious and time-consuming—but still necessary at this time (Fig. 4). One of the important factors contributing to the infectivity of SARS-CoV-2 and its wide transmission which differs from SARS-CoV is that SARS-CoV-2 efficiently utilizes *TMPRSS2* for entry into cells (1,35). Previous studies also reported eQTL (expression quantitative trait loci) variants in *TMPRSS2* that can be possible candidate disease modulators, resulting in higher *TMPRSS2* expression involving three intronic SNPs [rs2070788, rs9974589 and rs7364083; (36)]. If that proves to be the case, designing effective protease inhibitors directed against *TMPRSS2* might be a feasible drug discovery strategy (37). One limitation of DMS is the fact that variants characterized by this methodology are located in the ORF of the host genes. However, a number of genomic and immunological biomarkers for COVID-19 severity and mortality identified by large cohort GWAS and Phenome-wide association study (PheWAS) studies are located outside of ORFs. Examples include rs2271616 (*SLC6A20*), which showed a strong association with SARS-CoV-2 infection, rs35044562 (*LZTFL1*), which is a risk allele for severe COVID-19 and immunological determinant type I IFNs, which is essential for host defense against SARS-CoV-2 (31,38,39). A series of SNPs including *SLC6A20/LZTFL1* (rs35081325, rs73062389 and rs2531743), *DDP9* (*r*s2277732)*, IFNAR2* (rs13050728), *OAS3* (rs7310667), *STM2A* (rs622568), *KAT7* (rs9903642), *CCHCR1* (rs143334143), *IGF1* (rs10860891), *TMPRSS2* (rs2298661) and *ABO* (rs505922) were identified by previous studies (9,31,40,41), but using eight phenotypes outside of typical clinical phenotypes, which confirmed the results reported by previous GWAS studies and expanded COVID-19 phenotype definitions to reveal that variation in the Chr3p21 region modulates multiple aspects of COVID-19 susceptibility and severity (42). Of course, additional genes of interest could be investigated later by DMS, so the list of genes that might influence SARS-CoV-2 infectivity or clinical course could be expanded to include relevant genes beyond *ACE2* and *TMPRSS2*. A comprehensive strategy for the individualized treatment of COVID-19 patients could potentially be developed by integrating information from multiple platforms, such as data from GWAS, PheWAS and eQTLs with additional functional information, information such as that provided by DMS (43).

In summary, we have identified and validated DNA sequence variants that might potentially be clinically relevant for COVID-19 patient outcomes. Functional studies of those variants showed increased protein expression, which could result in undesired elevated susceptibility and severity while variants showing decreased protein expression might have protective effects. We have recently proposed the increased application of preemptive DNA sequence-based data in pharmacogenomics broadly, such as that applied during the Mayo Clinic Right 10K study (44). As preemptive genomic information becomes increasingly available, the methodology used in the present stud*y* could be implemented to study many additional clinically important genes beyond *ACE2* and *TMPRSS2*.

## Materials and Methods

### Generation of variant libraries

Promotor-less attB-*ACE2* and attB-*TMPRSS2* (isoforms 1 and 2) plasmids were created by Gibson Assembly using cDNA plasmids as described subsequently. Specifically, the C-terminal sequences of the *ACE2* or *TMPRSS2* (isoforms 1 and 2) ORFs were fused to GFP and mCherry, which served as markers for Bxb1 recombinase efficiency for integrating into the landing pad #20 cell line that we had established previously (22). That platform was designed to ensure only one variant per cell for downstream analysis. Specifically, human *ACE2* (NM_021804.2) ORF cDNA was obtained from Genscript (Piscataway, NJ) and the human *TMPRSS2* isoform1 (NM_001135099.1) ORF cDNA was obtained from Genscript (Piscataway, NJ), whereas the *TMPRSS2* isoform2 GFP cDNA (NM_005656.3) was obtained from Sino Biological (Beijing, China). Site-directed mutagenesis was used to construct variant libraries for ORFs containing *ACE2* and *TMPRSS2* (isoforms 1 and 2) missense variants. Primer oligonucleotides for *ACE2* and *TMPRSS2* variants were purchased from IDT (Coralville, IW). Sanger sequencing was used to validate the sequences of the variant clones. Pooled variants of attB-*ACE2* or attB-*TMPRSS2* promotor-less cassettes, respectively were transfected into the landing pad #20 platform. 24 h after transfection with Bxb1 recombinase, BFP in landing pad#20 was induced by doxycycline. After 5 days, the cells were trypsinized and washed with PBS for the downstream FACS sorting. Cells that successfully integrated variants of either gene, i.e. BFP^−^/mCherry^+^, were collected by FACSAria sorting (BD Biosciences, San Jose, California, United States) as pooled variant libraries. Flow cytometry was performed on FACS CantoX, which utilizes colinear 405, 488 and 561 nm lasers plus forward and side angle light scatter with the FACSDiva v8.0.1 software. The FACS sorting was performed on a FACSAria sorter with 407, 488 and 532 nm lasers (BD Biosciences). Data were analyzed by FACSDiva v8.0.1 software.

### Fluorescence —activated cell sorting

For the FACS sorting of *ACE2* or *TMPRSS2* protein expression, pooled variant cells (BFP^−^/mCherry^+^ cells) were washed, trypsinized and resuspended in PBS containing 2% FBS and 10 mm HEPES. BFP^−^/mCherry^+^ cells containing *ACE2* or *TMPRSS2* variants were flow sorted and grown in 10% FBS with DMEM culture medium with 2 μg/ml doxycycline for 7 days. BFP^−^/mCherry^+^ cells were then sorted again to determine the protein expression of *ACE2*/*TMPRSS2* variants based on their GFP/mCherry ratios. Gates were set based on GFP/mCherry ratios for wild-type proteins as gating references. Four gates were set to dissect the pooled libraries into four different bins based on GFP/mCherry ratios.

### Variant calling

Variant frequencies for *ACE2* or *TMPRSS2* variants were calculated by high-throughput sequencing of the DNA collected in each bin from the FACS analysis. Specifically, genomic DNA was extracted using DNA extraction kits (Qiagen, Germany) and amplicons were produced by PCR ﻿using KAPA HiFi HotStart ReadyMix (Kapa Biosystems, Willmington, MA). Primers were designed to bind to common non-mutated regions of the cassette sequences. The PCR products were purified by QIAquick Gel Purification Kit (Qiagen, Germany) and were quantified by use of the Qubit® dsDNA HS Reagent (Fisher Scientific, Hampton, NH). Amplicon DNA (1 ng) was used as the starting material for library preparation by use of the Nextera XT DNA Preparation Kit (Illumina, San Diego, CA). Samples used for library preparation were pooled after indexing and were sequenced with the Illumina HiSeq4000 Sequencing System in rapid run mode using the TruSeq Rapid SBS Kit (Illumina, San Diego, CA) with 300 cycle and 2X150bp paired-end reads capability. Fastq files were aligned with respective *ACE2* and *TMPRSS2* (isoforms 1 and 2) reference sequences using BWA mem aligner version 0.7.15. Samtools mpileup version 1.5 was used with a custom python script for SNV calling. A base quality score cut-off of 20 and a mapping quality score cut-off of 20 were applied for SNV calling. Custom scripts were used to summarize the data and to add allele frequencies at all positions in the reference sequence. Variant counts in each bin were tabulated and each variant’s frequency in each bin was calculated. The abundance scores for variants were obtained based on the frequency of variants in each bin.

### Western blots

Protein lysates of BFP^−^/mCherry^+^ cells containing individual variants for *ACE2* or the two *TMPRSS2* isoforms were lysed with M-PER™ buffer (ThermoFisher Scientific, Cat. No. 78501, Waltham, Massachusetts, United States). Proteins were separated by SDS-PAGE prior to transfer to PVDF membranes. Those membranes were incubated with rabbit polyclonal *ACE2* antibody (Santa Cruz, Cat. No. Sc-390 851, Dallas, Texas, United States) at a 1:1000 dilution or *TMPRSS2* rabbit monoclonal antibody (Abclonal, Cat. No. A9126, Woburn, Massachusetts, United States) at a 1:1000 dilution. mCherry protein was measured using mouse monoclonal mCherry antibody (Sigma, Cat. No. SAB2702291, St. Louis, Missouri, United States) and the cell membrane marker sodium potassium ATPase was measured using rabbit monoclonal antibody (Abcam, Cat. No. ab76020), and their expressions were used as loading controls. Proteins were detected using the Western Lightning Plus-ECL (Perkin Elmer, Cat. No. NEL104001EA, Waltham, Massachusetts, United States), and images were captured on X-ray film or by use of the ChemiDoc™ Touch Image System (Bio-Rad, Hercules, CA).

## Conflict of Interest statement

Both Drs Weinshilboum and Wang are co-founders of and stockholders in OneOme, LLC. The other authors have no conflicts to declare.

## Funding

National Institutes of Health grants U19 GM61388 (The Pharmacogenomics Research Network), R01 GM028157, R01GM125633, R01 AA027486, K01 AA28050; National Science Foundation Award IIS-2041339, and the Mayo ClinicCenter for Individualized Medicine.

## Authors’ contributions

L.Z., D. L., K.N.L., L.W. and R.W. participated in research design; L.Z., I.M. and M.H. conducted experiments; L.Z., A.A, V.S., L.X.W., A.R.G., R.A.V. and N.B.L performed data analysis; L.Z., D.L., L.W. and R.W. contributed to the writing of the manuscript; All authors have given final approval of the manuscript for submission.

## Supplementary Material

Supplementary_Table_S1_ddac157

Supplementary_Table_S2_ddac157

Supplementary_Table_S3__ddac157

Supplementary_Table_S4_ddac157

Supplementary_Table_S5_ddac157

Supplementary_Table_S6_ddac157
