# Supplementary material for: ACE2 and TMPRSS2 SARS-CoV-2 infectivity genes: deep mutational scanning and characterization of missense variants
Source: Hum Mol Genet. 2022 Jul 21;31(24):4183–92. doi: 10.1093/hmg/ddac157 (PMC9759330; doi:10.1093/hmg/ddac157)
Supplement: Supplementary_Table_S1_ddac157 [file supplementary_table_s1_ddac157.docx]

| Supplementary Table 1. Abundance score for ACE2 expression and prediction algorithm probabilities of ACE2 function | | | | | | | | |
| --- | --- | --- | --- | --- | --- | --- | --- | --- |
| EXACT cDNA | **EXACT Amino acid** | **RSID** | **Allele Frequency** | **SIFT** | **PROVEAN** | **PolyPhen2** | **CADD Phred Score** | **DMS abundance score** |
|  |  |  |  |  |  |  |  |  |
| c.2158A>G | p.Asn720Asp | rs41303171 | 0.01621501 | Tolerated | Neutral | neutral | 14.9 | 0.672442694 |
| c.77A>G | p.Lys26Arg | rs4646116 | 0.0038833 | Tolerated | Neutral | neutral | 8.188 | 0.625345493 |
| c.2191C>T | p.Leu731Phe | rs147311723 | 0.00143491 | Damaging | Neutral | deleterious | 22.7 | 0.596686504 |
| c.631G>A | p.Gly211Arg | rs148771870 | 0.00127989 | Tolerated | Neutral | deleterious | 15.59 | 0.733922102 |
| c.1402A>G | p.Ile468Val | rs191860450 | 0.00083844 | Tolerated | Neutral | deleterious | 24.8 | 0.648734004 |
| c.2074T>C | p.Ser692Pro | rs149039346 | 0.00056188 | Damaging | Neutral | deleterious | 14.9 | 0.599123292 |
| c.1022A>G | p.Lys341Arg | rs138390800 | 0.00040019 | Damaging | Neutral | neutral | 17.54 | 0.712025396 |
| c.655C>T | p.Arg219Cys | rs372272603 | 0.00034806 | Damaging | Neutral | deleterious | 23 | 0.651455026 |
| c.55T>C | p.Ser19Pro | rs73635825 | 0.00031292 | Tolerated | Neutral | deleterious | 16.16 | 0.697439654 |
| c.617A>G | p.Asp206Gly | rs142443432 | 0.0003 | Tolerated | Neutral | neutral | 21.3 | 0.624525049 |
| c.1913A>G | p.Asn638Ser | rs183135788 | 0.00025348 | Tolerated | Neutral | neutral | 14.22 | 0.736842105 |
| c.2089A>G | p.Arg697Gly | rs751603885 | 0.00025213 | Damaging | Neutral | deleterious | 16.96 | 0.686271676 |
| c.1640C>G | p.Ser547Cys | rs373025684 | 0.00021059 | Damaging | Deleterious | deleterious | 24.2 | 0.648118728 |
| c.1840G>T | p.Ala614Ser | rs201715513 | 0.00017115 | Tolerated | Neutral | neutral | 0.096 | 0.709834129 |
| c.344G>A | p.Arg115Gln | rs201900069 | 0.00017031 | Tolerated | Neutral | neutral | 0.877 | 0.570662607 |
| c.1791C>A | p.Asp597Glu | rs145437639 | 0.00012337 | Tolerated | Neutral | neutral | 0.001 | 0.701190476 |
| c.2221A>G | p.Ile741Val | rs372923812 | 0.00010035 | Tolerated | Neutral | neutral | 0.066 | 0.685184177 |
| c.656G>A | p.Arg219His | rs759590772 | 9.8313E-05 | Damaging | Neutral | deleterious | 23.5 | 0.615300863 |
| c.1133A>G | p.His378Arg | rs142984500 | 8.791E-05 | Damaging | Deleterious | deleterious | 25 | 0.665199565 |
| c.2147G>A | p.Arg716His | rs200540199 | 8.1803E-05 | Damaging | Neutral | deleterious | 11.52 | 0.665874907 |
| c.22C>T | p.Leu8Phe | rs201035388 | 8.0581E-05 | Tolerated | Neutral | neutral | 12.09 | 0.341416642 |
| c.1339G>T | p.Val447Phe | rs776328956 | 6.6844E-05 | Damaging | Deleterious | deleterious | 25.1 | 0.484375 |
| c.2345C>T | p.Ala782Val | rs147487891 | 6.0784E-05 | Tolerated | Neutral | neutral | 10.87 | 0.686221591 |
| c.2315A>G | p.Asn772Ser | rs764682532 | 6.021E-05 | Tolerated | Neutral | neutral | 0.065 | 0.651091457 |
| c.787C>T | p.Pro263Ser | rs200745906 | 5.7841E-05 | Damaging | Deleterious | deleterious | 25.8 | 0.561492387 |
| c.1594G>A | p.Ala532Thr | rs763593286 | 5.463E-05 | Tolerated | Deleterious | neutral | 15.61 | 0.535390878 |
| c.1481A>T | p.Asp494Val | rs765152220 | 4.9578E-05 | Damaging | Deleterious | deleterious | 31 | 0.72339221 |
| c.884A>G | p.Asp295Gly | rs776226831 | 4.6594E-05 | Damaging | Deleterious | neutral | 16.2 | 0.601153324 |
| c.1449G>C | p.Glu483Asp | rs779752560 | 4.6391E-05 | Tolerated | Neutral | neutral | 17.58 | 0.743925501 |
| c.1512C>A | p.Phe504Leu | rs1285805675 | 4.6307E-05 | Damaging | Deleterious | deleterious | 25.8 | 0.665495269 |
| c.2012G>C | p.Arg671Pro | rs753705431 | 4.6155E-05 | Tolerated | Neutral | neutral | 11.18 | 0.75 |
| c.1709T>C | p.Leu570Ser | rs1305384714 | 4.5988E-05 | Damaging | Deleterious | deleterious | 24.1 | 0.728759063 |
| c.2218T>C | p.Ser740Pro | rs1259850376 | 4.5975E-05 | Damaging | Neutral | neutral | 9.883 | 0.784689649 |
| c.551T>G | p.Val184Gly | rs758142853 | 4.5836E-05 | Damaging | Deleterious | deleterious | 28.8 | 0.656411511 |
| c.513G>C | p.Glu171Asp | rs1341206593 | 4.5552E-05 | Tolerated | Neutral | neutral | 10.14 | 0.595509495 |
| c.1961A>C | p.Tyr654Ser | rs1479485636 | 4.5521E-05 | Damaging | Deleterious | deleterious | 23.2 | 0.704100529 |
| c.2328T>A | p.Ser776Arg | rs1228927272 | 4.5502E-05 | Tolerated | Neutral | neutral | 10.49 | 0.656455009 |
| c.1258T>C | p.Ser420Pro | rs1384237654 | 4.5488E-05 | Damaging | Deleterious | neutral | 17.5 | 0.637386103 |
| c.1338T>G | p.Ile446Met | rs1290769028 | 4.5397E-05 | Tolerated | Neutral | neutral | 22.2 | 0.644037225 |
| c.2312A>G | p.Lys771Arg | rs1323382709 | 4.5376E-05 | Tolerated | Neutral | neutral | 12.31 | 0.621868032 |
| c.120C>G | p.Phe40Leu | rs924799658 | 4.5351E-05 | Tolerated | Neutral | neutral | 5.018 | 0.572916667 |
| c.551T>C | p.Val184Ala | rs758142853 | 4.365E-05 | Damaging | Deleterious | deleterious | 26.3 | 0.615959822 |
| c.2129G>A | p.Arg710His | rs370187012 | 3.9966E-05 | Damaging | Neutral | deleterious | 26.4 | 0.65280347 |
| c.1636A>G | p.Asn546Asp | rs761944150 | 3.9165E-05 | Damaging | Deleterious | deleterious | 22.2 | 0.642460317 |
| c.109G>A | p.Glu37Lys | rs146676783 | 3.8971E-05 | Tolerated | Neutral | deleterious | 23.7 | 0.546489993 |
| c.2353G>A | p.Asp785Asn | rs373153165 | 3.8668E-05 | Damaging | Neutral | neutral | 14.65 | 0.691409656 |
| c.1166C>A | p.Pro389His | rs762890235 | 3.8258E-05 | Damaging | Deleterious | deleterious | 24 | 0.610896702 |
| c.986A>G | p.Glu329Gly | rs143936283 | 3.443E-05 | Tolerated | Neutral | neutral | 12.04 | 0.680929152 |
| c.808A>G | p.Met270Val | rs766319182 | 2.983E-05 | Damaging | Deleterious | deleterious | 24.8 | 0.619651442 |
| c.2128C>T | p.Arg710Cys | rs901495523 | 2.8919E-05 | Damaging | Deleterious | deleterious | 27.4 | 0.628470795 |
| c.1844A>G | p.Asp615Gly | rs773083611 | 2.7393E-05 | Tolerated | Neutral | neutral | 16.52 | 0.639032101 |
| c.770G>A | p.Ser257Asn | rs745514718 | 2.5705E-05 | Tolerated | Neutral | neutral | 0.047 | 0.629569804 |
| c.246G>A | p.Met82Ile | rs766996587 | 2.4418E-05 | Tolerated | Neutral | neutral | 0.027 | 0.674908938 |
| c.2413T>A | p.Phe805Ile | rs749525058 | 2.3209E-05 | Damaging | Neutral | deleterious | 21.8 | 0.583932025 |
| c.1399G>A | p.Glu467Lys | rs1270795706 | 2.2425E-05 | Tolerated | Neutral | neutral | 22.2 | 0.715902909 |
| c.1501G>A | p.Ala501Thr | rs140473595 | 2.2166E-05 | Damaging | Neutral | deleterious | 23.6 | 0.627195025 |
| c.2002G>A | p.Glu668Lys | rs200180615 | 2.1997E-05 | Tolerated | Neutral | neutral | 13.35 | 0.618500535 |
| c.1163A>T | p.Gln388Leu | rs751572714 | 2.1861E-05 | Tolerated | Neutral | neutral | 19.53 | 0.584543327 |
| c.517G>A | p.Gly173Ser | rs754511501 | 2.1817E-05 | Damaging | Deleterious | deleterious | 26.2 | 0.735378927 |
| c.2012G>A | p.Arg671Gln | rs753705431 | 1.9619E-05 | Tolerated | Neutral | neutral | 10.19 | 0.737374409 |
| c.2122C>T | p.Arg708Trp | rs776995986 | 1.804E-05 | Damaging | Deleterious | deleterious | 22.9 | 0.666931464 |
| c.1445G>A | p.Arg482Gln | rs748359955 | 1.7609E-05 | Tolerated | Neutral | deleterious | 26.3 | 0.587654684 |
| c.725C>T | p.Ala242Val | rs1360329820 | 1.7254E-05 | Damaging | Deleterious | deleterious | 27 | 0.562373191 |
| c.1825G>A | p.Asp609Asn | rs747988885 | 1.7186E-05 | Tolerated | Neutral | neutral | 4.349 | 0.646321916 |
| c.872T>A | p.Ile291Lys | rs756358940 | 1.7059E-05 | Damaging | Deleterious | deleterious | 26.6 | 0.66260498 |
| c.596A>G | p.Tyr199Cys | rs750145841 | 1.6878E-05 | Tolerated | Deleterious | deleterious | 26.2 | 0.614182692 |
| c.1013A>G | p.Asn338Ser | rs916715573 | 1.6594E-05 | Damaging | Neutral | neutral | 17.22 | 0.670207855 |
| c.2107G>T | p.Ala703Ser | rs780128908 | 1.6562E-05 | Damaging | Neutral | deleterious | 24 | 0.700312822 |
| c.994A>T | p.Met332Leu | rs185525294 | 1.6543E-05 | Damaging | Deleterious | deleterious | 19.32 | 0.718819561 |
| c.1783C>G | p.Leu595Val | rs148036434 | 1.6541E-05 | Damaging | Deleterious | deleterious | 25.1 | 0.551545707 |
| c.1745G>A | p.Arg582Lys | rs150172355 | 1.6435E-05 | Tolerated | Neutral | neutral | 0.001 | 0.680675575 |
| c.1888G>C | p.Asp630His | rs140312271 | 1.6424E-05 | Damaging | Neutral | deleterious | 20.8 | 0.658816324 |
| c.1125G>T | p.Glu375Asp | rs1395782023 | 1.6421E-05 | Damaging | Deleterious | deleterious | 25.8 | 0.780443572 |
| c.578C>A | p.Ala193Glu | rs762219565 | 1.6413E-05 | Tolerated | Neutral | neutral | 14.83 | 0.641465961 |
| c.2065A>G | p.Lys689Glu | rs777410473 | 1.6405E-05 | Tolerated | Neutral | neutral | 0.25 | 0.669401322 |
| c.658G>A | p.Gly220Ser | rs774621083 | 1.6389E-05 | Tolerated | Neutral | neutral | 1.265 | 0.681279376 |
| c.120C>A | p.Phe40Leu | rs924799658 | 1.6373E-05 | Tolerated | Neutral | neutral | 5.756 | 0.680291508 |
| c.476A>G | p.Asn159Ser | rs746034076 | 1.6368E-05 | Tolerated | Neutral | neutral | 0.005 | 0.684781365 |
| c.103G>A | p.Glu35Lys | rs1348114695 | 1.636E-05 | Tolerated | Neutral | neutral | 4.5 | 0.622800341 |
| c.2341T>C | p.Tyr781His | rs902013280 | 1.4797E-05 | Damaging | Neutral | deleterious | 24.9 | 0.571875 |
| c.305A>C | p.Gln102Pro | rs1395878099 | 1.4745E-05 | Tolerated | Neutral | neutral | 20.8 | 0.567455247 |
| c.1778C>A | p.Thr593Asn | rs140857723 | 1.4744E-05 | Tolerated | Neutral | neutral | 11.24 | 0.644418973 |
| c.648C>A | p.Asp216Glu | rs753164828 | 1.4705E-05 | Tolerated | Neutral | neutral | 0.098 | 0.589880952 |
| c.1746G>T | p.Arg582Ser | rs372924787 | 1.468E-05 | Tolerated | Neutral | neutral | 0.483 | 0.674934573 |
| c.192T>A | p.Asn64Lys | rs1199100713 | 1.4664E-05 | Tolerated | Neutral | neutral | 0.004 | 0.670702561 |
| c.1189A>G | p.Asn397Asp | rs1365935088 | 1.4641E-05 | Damaging | Deleterious | deleterious | 26.3 | 0.727915162 |
| c.2302C>T | p.Arg768Trp | rs140016715 | 1.3852E-05 | Damaging | Deleterious | deleterious | 24.2 | 0.6078125 |
| c.2299G>C | p.Asp767His | rs747516757 | 1.3428E-05 | Damaging | Neutral | deleterious | 16.9 | 0.688112776 |
| c.1510T>A | p.Phe504Ile | rs760281053 | 1.2682E-05 | Damaging | Deleterious | deleterious | 25.9 | 0.647277268 |
| c.907G>A | p.Asp303Asn | rs749750821 | 1.2027E-05 | Tolerated | Neutral | neutral | 7.936 | 0.834676836 |
| c.899A>G | p.Gln300Arg | rs773936807 | 1.1869E-05 | Damaging | Deleterious | deleterious | 32 | 0.64168668 |
| c.1063G>A | p.Asp355Asn | rs961360700 | 1.1743E-05 | Damaging | Deleterious | deleterious | 23.7 | 0.66865942 |
| c.2252G>A | p.Gly751Glu | rs761405491 | 1.1659E-05 | Damaging | Neutral | deleterious | 21.1 | 0.643272809 |
| c.2176G>C | p.Gly726Arg | rs139980377 | 1.1529E-05 | Damaging | Deleterious | deleterious | 25.5 | 0.717758625 |
| c.874G>A | p.Asp292Asn | rs1248745003 | 1.1397E-05 | Damaging | Deleterious | deleterious | 29.6 | 0.398146645 |
| c.934G>A | p.Glu312Lys | rs780574871 | 1.1341E-05 | Damaging | Deleterious | deleterious | 25 | 0.593707884 |
| c.868A>C | p.Asn290His | rs763994205 | 1.1332E-05 | Damaging | Deleterious | deleterious | 24.7 | 0.628125 |
| c.755A>G | p.Tyr252Cys | rs771769548 | 1.1283E-05 | Damaging | Deleterious | deleterious | 24.8 | 0.503579754 |
| c.2201C>T | p.Pro734Leu | rs751484521 | 1.1272E-05 | Damaging | Deleterious | deleterious | 24.7 | 0.764483853 |
| c.179A>G | p.Gln60Arg | rs759162332 | 1.1245E-05 | Tolerated | Neutral | neutral | 15.34 | 0.75 |
| c.172A>C | p.Asn58His | rs1222417695 | 1.117E-05 | Damaging | Deleterious | deleterious | 24.1 | 0.752604167 |
| c.2387A>G | p.Gln796Arg | rs1355591379 | 1.1132E-05 | Damaging | Neutral | deleterious | 16.22 | 0.635152348 |
| c.320T>C | p.Val107Ala | rs139773121 | 1.1055E-05 | Tolerated | Neutral | neutral | 0.003 | 0.640228824 |
| c.1097T>C | p.Met366Thr | rs758568640 | 1.0984E-05 | Damaging | Deleterious | deleterious | 24.2 | 0.587778236 |
| c.1718T>C | p.Val573Ala | rs1303528080 | 1.0981E-05 | Tolerated | Deleterious | neutral | 14.5 | 0.680700036 |
| c.2014G>C | p.Val672Leu | rs763939235 | 1.0971E-05 | Damaging | Neutral | neutral | 14 | 0.732638889 |
| c.2086C>A | p.Pro696Thr | rs755445931 | 1.0961E-05 | Damaging | Deleterious | deleterious | 23.1 | 0.709114884 |
| c.275C>T | p.Thr92Ile | rs763395248 | 1.0956E-05 | Tolerated | Deleterious | neutral | 4.068 | 0.632578291 |
| c.462C>G | p.Asn154Lys | rs1435872603 | 1.0954E-05 | Tolerated | Neutral | neutral | 0.014 | 0.641074754 |
| c.1880C>T | p.Ala627Val | rs748163894 | 1.0948E-05 | Damaging | Neutral | deleterious | 26.2 | 0.59125 |
| c.1279G>T | p.Asp427Tyr | rs1316056737 | 1.0948E-05 | Damaging | Deleterious | deleterious | 22.9 | 0.524431428 |
| c.202A>G | p.Lys68Glu | rs755691167 | 1.0946E-05 | Tolerated | Neutral | neutral | 12.87 | 0.680835053 |
| c.257A>G | p.Gln86Arg | rs746808776 | 1.0941E-05 | Tolerated | Neutral | neutral | 10.78 | 0.70014881 |
| c.61A>G | p.Ile21Val | rs778030746 | 1.0927E-05 | Tolerated | Neutral | neutral | 0.006 | 0.690724206 |
| c.79A>G | p.Thr27Ala | rs781255386 | 1.091E-05 | Tolerated | Neutral | neutral | 7.955 | 0.669234045 |
| c.776T>C | p.Ile259Thr | rs1161338919 | 1.0307E-05 | Tolerated | Neutral | neutral | 0.469 | 0.606971154 |
| c.2233G>A | p.Val745Ile | rs772641644 | 1.0129E-05 | Tolerated | Neutral | neutral | 12.39 | 0.642925963 |
| c.2186C>T | p.Pro729Leu | rs375923132 | 1.0084E-05 | Damaging | Deleterious | neutral | 22.4 | 0.732638889 |
| c.2411C>T | p.Ser804Phe | rs771107251 | 4.7345E-05 | Damaging | Neutral | deleterious | 24.2 | 0.705362852 |
| c.1055G>T | p.Gly352Val | rs370610075 | 3.8005E-05 | Tolerated | Deleterious | deleterious | 24.6 | 0.625075174 |
| c.977G>A | p.Gly326Glu | rs759579097 | 3.7927E-05 | Tolerated | Neutral | neutral | 12.28 | 0.71981052 |
| c.1540C>G | p.Arg514Gly |  | 9.5939E-06 | Damaging | Deleterious | deleterious | 27.7 | 0.644886364 |
| c.343C>T | p.Arg115Trp | rs1292756480 | 9.493E-06 | Damaging | Deleterious | deleterious | 23.7 | 0.599201858 |
| c.1677G>C | p.Arg559Ser | rs1016777825 | 9.4893E-06 | Tolerated | Neutral | neutral | 0.001 | 0.816225962 |
| c.751G>A | p.Ala251Thr | rs886466413 | 9.4823E-06 | Tolerated | Neutral | neutral | 3.276 | 0.704438773 |
| c.1343G>A | p.Met462Ile | rs763655186 | 9.4559E-06 | Damaging | Deleterious | deleterious | 25.3 | 0.574993824 |
| c.1658A>C | p.Lys553Thr | rs899455705 | 9.4333E-06 | Tolerated | Deleterious | neutral | 17.34 | 0.668134544 |
