# Supplementary material for: ACE2 and TMPRSS2 SARS-CoV-2 infectivity genes: deep mutational scanning and characterization of missense variants
Source: Hum Mol Genet. 2022 Jul 21;31(24):4183–92. doi: 10.1093/hmg/ddac157 (PMC9759330; doi:10.1093/hmg/ddac157)
Supplement: Supplementary_Table_S2_ddac157 [file supplementary_table_s2_ddac157.docx]

| Supplementary Table 2. Abundance score for TMPRSS2 isoform 1 and 2 protein expression and prediction algorithm probabilities of TMPRSS2 function | | | | | | | | | | |
| --- | --- | --- | --- | --- | --- | --- | --- | --- | --- | --- |
| Position | **RSID** | **Allele Frequency** | **SIFT** | **PROVEAN** | **PolyPhen2** | **CADD Phred Score** | **DMS abundance score** | |  |  |
|  |  |  |  |  |  |  | **isoform1** | **isoform2** |  |  |
| Chr21:42879909 | rs75603675 | 0.35059329 | Tolerated | Neutral | neutral | 11.6 | 0.549355159 | NA |  |  |
| Chr21:42852497 | rs12329760 | 0.24881585 | Damaging | Neutral | deleterious | 24.8 | 0.423064647 | 0.416272523 |  |  |
| Chr21:42866297 | rs61735793 | 0.00730817 | Tolerated | Neutral | neutral | 8.751 | 0.623268398 | 0.663194444 |  |  |
| Chr21:42866439 | rs61735791 | 0.00170174 | Tolerated | Neutral | neutral | 0.252 | 0.342013889 | 0.615936175 |  |  |
| Chr21:42866468 | rs61735790 | 0.00093202 | Tolerated | Deleterious | neutral | 15.96 | 0.664944591 | 0.604005029 |  |  |
| Chr21:42840394 | rs148125094 | 0.00089092 | Tolerated | Neutral | neutral | 7.914 | 0.825197307 | NA |  |  |
| Chr21:42866301 | rs114363287 | 0.00062593 | Tolerated | Neutral | deleterious | 12.14 | 0.643830128 | 0.659286348 |  |  |
| Chr21:42861487 | rs147711290 | 0.00057288 | Damaging | Deleterious | deleterious | 23 | 0.494645979 | 0.639914773 |  |  |
| Chr21:42866388 | rs201679623 | 0.00048129 | Tolerated | Deleterious | deleterious | 25 | 0.272781971 | 0.644406703 |  |  |
| Chr21:42851157 | rs150554820 | 0.00043841 | Damaging | Deleterious | deleterious | 22.5 | 0.665462028 | 0.514344058 |  |  |
| Chr21:42845373 | rs61735796 | 0.00038761 | Tolerated | Neutral | neutral | 1.972 | 0.521309759 | 0.615388878 |  |  |
| Chr21:42866399 | rs138651919 | 0.00032554 | Damaging | Deleterious | deleterious | 18.11 | 0.604406802 | 0.652037607 |  |  |
| Chr21:42842623 | rs61735795 | 0.00031609 | Tolerated | Deleterious | neutral | 12.16 | 0.678034061 | 0.451683438 |  |  |
| Chr21:42845313 | rs142446494 | 0.00017459 | Damaging | Neutral | deleterious | 20.9 | 0.713443772 | 0.680803571 |  |  |
| Chr21:42866423 | rs201093031 | 0.00016625 | Tolerated | Neutral | neutral | 0.328 | 0.679557482 | 0.580356491 |  |  |
| Chr21:42843882 | rs768173297 | 0.00015639 | Tolerated | Neutral | deleterious | 14.14 | 0.655329099 | 0.674509804 |  |  |
| Chr21:42843822 | rs565237319 | 0.00015576 | Tolerated | Deleterious | neutral | 9.157 | 0.641071429 | 0.591567011 |  |  |
| Chr21:42866396 | rs376143876 | 0.00015521 | Damaging | Neutral | neutral | 14.35 | 0.555059686 | 0.639904898 |  |  |
| Chr21:42848546 | rs143597099 | 0.00015149 | Tolerated | Deleterious | neutral | 15.91 | NA | 0.534291444 |  |  |
| Chr21:42866301 | rs114363287 | 0.00014029 | Tolerated | Neutral | neutral | 11.45 | 0.723665398 | NA |  |  |
| Chr21:42845366 | rs150445636 | 0.00013531 | Tolerated | Deleterious | deleterious | 23.1 | 0.688596485 | 0.560801091 |  |  |
| Chr21:42861487 | rs147711290 | 0.00011934 | Damaging | Deleterious | deleterious | 23 | 0.59629206 | 0.613054061 |  |  |
| Chr21:42866328 | rs150389990 | 0.00010745 | Tolerated | Neutral | neutral | 0.004 | 0.70154533 | NA |  |  |
| Chr21:42848536 | rs547544037 | 9.5847E-05 | Tolerated | Neutral | neutral | 22.1 | 0.769290995 | 0.326041667 |  |  |
| Chr21:42879886 | rs1243033377 | 9.5767E-05 | Damaging | Neutral | neutral | 2.44 | 0.714285888 | NA |  |  |
| Chr21:42860333 | rs758778273 | 7.56E-05 | Damaging | Deleterious | deleterious | 26.2 | 0.617162936 | 0.609375 |  |  |
| Chr21:42845360 | rs775494034 | 7.4318E-05 | Tolerated | Neutral | neutral | 0.001 | 0.552070013 | 0.622223339 |  |  |
| Chr21:42843896 | rs1347220628 | 6.3739E-05 | Tolerated | Neutral | neutral | 0.388 | NA | 0.560449286 |  |  |
| Chr21:42839729 | rs1179096287 | 6.371E-05 | Tolerated | Neutral | neutral | 9.659 | 0.751910127 | 0.878125 |  |  |
| Chr21:42866348 | rs770214639 | 6.0609E-05 | Damaging | Deleterious | deleterious | 22.3 | 0.635833876 | 0.532326681 |  |  |
| Chr21:42843873 | rs748528218 | 6.038E-05 | Tolerated | Deleterious | neutral | 10.05 | 0.675717753 | 0.638983769 |  |  |
| Chr21:42852452 | rs748571451 | 5.9838E-05 | Tolerated | Neutral | neutral | 8.109 | 0.64698223 | 0.75 |  |  |
| Chr21:42843810 | rs200744510 | 5.6779E-05 | Tolerated | Neutral | deleterious | 16.69 | 0.654333472 | 0.676139699 |  |  |
| Chr21:42845367 | rs757466150 | 5.1276E-05 | Damaging | Deleterious | deleterious | 22.9 | 0.707291667 | 0.66889881 |  |  |
| Chr21:42851202 | rs139926880 | 4.9658E-05 | Tolerated | Deleterious | deleterious | 23.3 | 0.688096045 | 0.609411096 |  |  |
| Chr21:42861476 | rs190265904 | 4.7734E-05 | Damaging | Neutral | deleterious | 19.96 | 0.724744342 | NA |  |  |
| Chr21:42845259 | rs747772174 | 4.2121E-05 | Damaging | Neutral | deleterious | 24.2 | 0.675260829 | 0.675436466 |  |  |
| Chr21:42848539 | rs944739499 | 4.1722E-05 | Damaging | Deleterious | deleterious | 23.5 | 0.723545122 | NA |  |  |
| Chr21:42866331 | rs574582815 | 4.0387E-05 | Tolerated | Neutral | neutral | 4.867 | 0.281814139 | 0.4375 |  |  |
| Chr21:42845396 | rs775404304 | 4.036E-05 | Tolerated | Neutral | neutral | 10.1 | 0.550290257 | 0.695740274 |  |  |
| Chr21:42843887 | rs769164587 | 4.0019E-05 | Tolerated | Neutral | neutral | 0.004 | 0.606674944 | 0.623322427 |  |  |
| Chr21:42843823 | rs775137340 | 3.994E-05 | Tolerated | Neutral | neutral | 11.25 | 0.415178571 | 0.580717553 |  |  |
| Chr21:42843843 | rs144046631 | 3.9038E-05 | Tolerated | Deleterious | deleterious | 22.8 | 0.67872369 | 0.644628549 |  |  |
| Chr21:42866442 | rs554868303 | 3.8912E-05 | Tolerated | Neutral | neutral | 0.003 | NA | 0.731081192 |  |  |
| Chr21:42861463 | rs201949634 | 3.891E-05 | Tolerated | Neutral | neutral | 2.744 | 0.583866901 | 0.60321831 |  |  |
| Chr21:42848512 | rs760039204 | 3.589E-05 | Tolerated | Deleterious | deleterious | 22.3 | 0.718269981 | 0.691193182 |  |  |
| Chr21:42866456 | rs150502923 | 3.5848E-05 | Damaging | Deleterious | neutral | 19.45 | 0.633712121 | 0.645495139 |  |  |
| Chr21:42845397 | rs762854045 | 3.3149E-05 | Tolerated | Neutral | deleterious | 15.54 | 0.747160948 | 0.55 |  |  |
| Chr21:42866283 | rs779085411 | 3.2218E-05 | Damaging | Neutral | deleterious | 22.6 | 0.66214364 | 0.622916667 |  |  |
| Chr21:42879925 | rs1299493587 | 3.1959E-05 | Damaging | Neutral | neutral | 2.453 | 0.333285224 | NA |  |  |
| Chr21:42866495 | rs767591322 | 3.1923E-05 | Damaging | Deleterious | deleterious | 22.7 | 0.631903375 | 0.66211968 |  |  |
| Chr21:42879880 | rs916319861 | 3.191E-05 | Tolerated | Neutral | deleterious | 9.193 | 0.663134245 | NA |  |  |
| Chr21:42879900 | rs1406664235 | 3.1908E-05 | Tolerated | Neutral | deleterious | 9.831 | 0.700789571 | NA |  |  |
| Chr21:42866505 | rs1466538703 | 3.1896E-05 | Damaging | Deleterious | deleterious | 24.2 | 0.273772888 | 0.652627139 |  |  |
| Chr21:42852407 | rs1401630535 | 3.1884E-05 | Damaging | Deleterious | deleterious | 24.6 | 0.791332952 | 0.467210526 |  |  |
| Chr21:42866424 | rs1387038867 | 3.1871E-05 | Tolerated | Neutral | neutral | 6.504 | 0.690187278 | 0.631510417 |  |  |
| Chr21:42866411 | rs1175715553 | 3.1869E-05 | Damaging | Deleterious | deleterious | 19.45 | 0.506924934 | 0.572243283 |  |  |
| Chr21:42866391 | rs547639377 | 3.1865E-05 | Damaging | Deleterious | deleterious | 16.56 | 0.4625 | NA |  |  |
| Chr21:42842656 | rs1430677365 | 3.1861E-05 | Damaging | Deleterious | deleterious | 24 | 0.457810656 | 0.711525069 |  |  |
| Chr21:42839744 | rs1472953828 | 3.1859E-05 | Damaging | Deleterious | deleterious | 25 | 0.704096031 | 0.614956962 |  |  |
| Chr21:42840325 | rs772900547 | 3.1859E-05 | Damaging | Deleterious | deleterious | 25.8 | 0.70566313 | 0.639899381 |  |  |
| Chr21:42866328 | rs150389990 | 3.1859E-05 | Damaging | Neutral | deleterious | 11.35 | 0.584691356 | 0.626452823 |  |  |
| Chr21:42839743 | rs936556491 | 3.1855E-05 | Damaging | Deleterious | deleterious | 24.7 | 0.71168914 | 0.654612432 |  |  |
| Chr21:42840396 | rs1415259360 | 3.1855E-05 | Tolerated | Deleterious | deleterious | 15.44 | 0.680913865 | 0.628453407 |  |  |
| Chr21:42861490 | rs1350156643 | 3.1855E-05 | Tolerated | Neutral | neutral | 5.473 | 0.651514202 | 0.685814386 |  |  |
| Chr21:42860428 | rs1233717608 | 3.1851E-05 | Damaging | Deleterious | deleterious | 23.2 | 0.698918269 | 0.627403846 |  |  |
| Chr21:42839774 | rs1166094493 | 3.1849E-05 | Tolerated | Neutral | neutral | 0.001 | 0.476256316 | 0.589668669 |  |  |
| Chr21:42839719 | rs368268847 | 3.1847E-05 | Damaging | Deleterious | deleterious | 23.7 | 0.710646518 | 0.609063839 |  |  |
| Chr21:42870068 | rs1331618004 | 3.1847E-05 | Tolerated | Neutral | neutral | 6.018 | 0.593754064 | NA |  |  |
| Chr21:42843804 | rs1185182900 | 3.1845E-05 | Damaging | Deleterious | deleterious | 22.6 | 0.705952381 | 0.544886364 |  |  |
| Chr21:42866343 | rs1266959483 | 3.1845E-05 | Tolerated | Neutral | neutral | 0.922 | 0.662676156 | 0.695811124 |  |  |
| Chr21:42860345 | rs1385130606 | 3.1843E-05 | Tolerated | Neutral | neutral | 0.125 | 0.726188568 | 0.701347233 |  |  |
| Chr21:42845268 | rs372563970 | 2.987E-05 | Damaging | Deleterious | deleterious | 25.2 | 0.768905397 | 0.758287147 |  |  |
| Chr21:42845277 | rs776538081 | 2.9581E-05 | Damaging | Neutral | deleterious | 22.3 | 0.702880609 | 0.611201299 |  |  |
| Chr21:42845328 | rs140547429 | 2.8786E-05 | Tolerated | Neutral | deleterious | 17.67 | 0.677757972 | 0.693575384 |  |  |
| Chr21:42842590 | rs369579311 | 2.8463E-05 | Tolerated | Neutral | neutral | 20.2 | 0.595061278 | 0.697783605 |  |  |
| Chr21:42866376 | rs777667088 | 2.8363E-05 | Damaging | Neutral | deleterious | 20.3 | 0.654805248 | 0.641959752 |  |  |
| Chr21:42866364 | rs753509316 | 2.7998E-05 | Tolerated | Neutral | neutral | 0.001 | 0.649372569 | 0.666374868 |  |  |
| Chr21:42866409 | rs762928261 | 2.7855E-05 | Tolerated | Neutral | deleterious | 0.085 | 0.630303724 | 0.698958333 |  |  |
| Chr21:42843906 | rs561063944 | 2.5355E-05 | Tolerated | Deleterious | neutral | 21.3 | 0.700022583 | 0.624796063 |  |  |
| Chr21:42845376 | rs537370123 | 2.4804E-05 | Damaging | Deleterious | deleterious | 23.9 | 0.551420993 | 0.639622368 |  |  |
| Chr21:42866360 | rs766068032 | 2.4002E-05 | Damaging | Deleterious | deleterious | 23.6 | 0.71040029 | 0.562682749 |  |  |
| Chr21:42843768 | rs745742232 | 2.3989E-05 | Damaging | Deleterious | deleterious | 24.8 | 0.762315495 | 0.658004764 |  |  |
| Chr21:42866369 | rs752650649 | 2.3936E-05 | Tolerated | Neutral | neutral | 18.25 | 0.470982143 | 0.622660617 |  |  |
| Chr21:42839686 | rs541351488 | 2.3874E-05 | Tolerated | Neutral | deleterious | 16.04 | 0.553893098 | 0.607391154 |  |  |
| Chr21:42860405 | rs199865751 | 2.1252E-05 | Tolerated | Neutral | neutral | 14.77 | 0.619249561 | 0.659864364 |  |  |
| Chr21:42866414 | rs141232947 | 2.1225E-05 | Tolerated | Neutral | neutral | 0.002 | 0.711595249 | 0.685847454 |  |  |
| Chr21:42861500 | rs763645871 | 2.1222E-05 | Tolerated | Neutral | neutral | 0.001 | 0.794270833 | 0.623787032 |  |  |
| Chr21:42845286 | rs530689404 | 2.0696E-05 | Tolerated | Neutral | neutral | 15.57 | 0.682677926 | 0.534472675 |  |  |
| Chr21:42860422 | rs142988104 | 1.9975E-05 | Tolerated | Neutral | neutral | 0.001 | 0.615603646 | 0.556442308 |  |  |
| Chr21:42843832 | rs757214557 | 1.9971E-05 | Tolerated | Neutral | neutral | 0.001 | 0.521581959 | 0.637691962 |  |  |
| Chr21:42866477 | rs774327563 | 1.9945E-05 | Tolerated | Deleterious | neutral | 21.9 | 0.711231338 | 0.470486111 |  |  |
| Chr21:42852443 | rs139092674 | 1.9945E-05 | Tolerated | Deleterious | deleterious | 18.28 | 0.615384615 | 0.663236873 |  |  |
| Chr21:42840465 | rs762844469 | 1.9897E-05 | Damaging | Deleterious | deleterious | 33 | 0.692325431 | 0.601964722 |  |  |
| Chr21:42866331 | rs574582815 | 1.7922E-05 | Damaging | Neutral | neutral | 2.047 | 0.623960197 | 0.625 |  |  |
| Chr21:42842584 | rs147233451 | 1.7789E-05 | Tolerated | Neutral | neutral | 11.04 | 0.586231353 | 0.606659831 |  |  |
| Chr21:42861434 | rs368936645 | 1.7697E-05 | Tolerated | Neutral | neutral | 3.057 | 0.605365535 | 0.609665888 |  |  |
| Chr21:42840423 | rs746555310 | 1.7677E-05 | Damaging | Deleterious | deleterious | 23.4 | 0.685654023 | 0.588839635 |  |  |
| Chr21:42879898 | rs765381251 | 1.6715E-05 | Damaging | Neutral | neutral | 6.362 | 0.753795721 | NA |  |  |
| Chr21:42845291 | rs764393597 | 1.6479E-05 | Damaging | Deleterious | deleterious | 23.9 | 0.645318602 | 0.644683493 |  |  |
| Chr21:42866336 | rs771209150 | 1.6134E-05 | Tolerated | Neutral | neutral | 1.24 | 0.692025747 | 0.659722222 |  |  |
| Chr21:42866342 | rs377060358 | 1.6106E-05 | Damaging | Neutral | deleterious | 9.16 | 0.700961643 | 0.628472222 |  |  |
| Chr21:42843894 | rs540987630 | 1.6039E-05 | Damaging | Deleterious | deleterious | 22.4 | 0.588909775 | 0.58073774 |  |  |
| Chr21:42852446 | rs373847134 | 1.5957E-05 | Damaging | Deleterious | deleterious | 23.7 | 0.702265091 | 0.578096964 |  |  |
| Chr21:42848513 | rs762108701 | 1.5951E-05 | Damaging | Deleterious | deleterious | 25.9 | 0.606796117 | 0.672609187 |  |  |
| Chr21:42852481 | rs759250613 | 1.5945E-05 | Tolerated | Neutral | neutral | 18.79 | 0.671875 | 0.552083333 |  |  |
| Chr21:42860360 | rs760565628 | 1.5915E-05 | Damaging | Neutral | deleterious | 16.07 | 0.644632711 | 0.666892058 |  |  |
| Chr21:42851120 | rs148049486 | 1.5914E-05 | Damaging | Neutral | deleterious | 19.44 | 0.676459543 | 0.644586784 |  |  |
| Chr21:42861481 | rs761195761 | 1.5912E-05 | Tolerated | Neutral | neutral | 5.014 | 0.568452381 | NA |  |  |
| Chr21:42842599 | rs755712060 | 1.4216E-05 | Damaging | Deleterious | deleterious | 26.7 | 0.756609603 | 0.663936546 |  |  |
| Chr21:42870050 | rs763515247 | 1.4191E-05 | Tolerated | Neutral | neutral | 18.46 | 0.7625 | 0.722054576 |  |  |
| Chr21:42852453 | rs376158219 | 1.4181E-05 | Tolerated | Deleterious | deleterious | 2.949 | 0.545077652 | 0.658241758 |  |  |
| Chr21:42852427 | rs751523924 | 1.4178E-05 | Tolerated | Neutral | deleterious | 11.57 | 0.272328785 | 0.389636752 |  |  |
| Chr21:42866405 | rs775450506 | 1.4153E-05 | Tolerated | Neutral | deleterious | 0.333 | 0.55 | 0.551395757 |  |  |
| Chr21:42866441 | rs770444484 | 1.4149E-05 | Damaging | Deleterious | deleterious | 17.55 | NA | 0.708291997 |  |  |
| Chr21:42839785 | rs773001010 | 1.4141E-05 | Tolerated | Neutral | neutral | 0.009 | 0.551081731 | 0.616579239 |  |  |
| Chr21:42845267 | rs146654734 | 1.2776E-05 | Damaging | Deleterious | deleterious | 24.2 | 0.661323052 | 0.569444444 |  |  |
| Chr21:42845397 | rs762854045 | 1.2431E-05 | Tolerated | Neutral | neutral | 2.259 | 0.609009587 | 0.660258862 |  |  |
| Chr21:42845307 | rs757634613 | 1.2316E-05 | Damaging | Deleterious | deleterious | 26.5 | 0.683335566 | 0.636941964 |  |  |
| Chr21:42843733 | rs758128660 | 1.221E-05 | Tolerated | Neutral | neutral | 23.6 | 0.522099651 | 0.619965882 |  |  |
| Chr21:42866307 | rs200169208 | 1.2193E-05 | Tolerated | Neutral | neutral | 3.645 | 0.760625702 | 0.594163383 |  |  |
| Chr21:42860437 | rs771653895 | 1.2071E-05 | Tolerated | Neutral | neutral | 5.189 | 0.632869472 | 0.619466422 |  |  |
| Chr21:42843874 | rs772504668 | 1.1991E-05 | Damaging | Deleterious | deleterious | 22.5 | 0.605585831 | 0.597458818 |  |  |
| Chr21:42843808 | rs772196502 | 1.1982E-05 | Damaging | Deleterious | deleterious | 22.4 | 0.676913496 | 0.702969615 |  |  |
| Chr21:42852434 | rs781089181 | 1.1967E-05 | Damaging | Deleterious | deleterious | 23 | 0.586749832 | 0.727469239 |  |  |
| Chr21:42860412 | rs751035521 | 1.1962E-05 | Tolerated | Neutral | neutral | 0.015 | 0.469536393 | 0.629283371 |  |  |
| Chr21:42852518 | rs766503231 | 1.196E-05 | Damaging | Deleterious | deleterious | 27 | 0.666666667 | 0.533630952 |  |  |
| Chr21:42852504 | rs756213944 | 1.1956E-05 | Tolerated | Neutral | neutral | 16.26 | 0.460596324 | 0.617370064 |  |  |
| Chr21:42866381 | rs746151019 | 1.1953E-05 | Damaging | Deleterious | deleterious | 23 | 0.623378919 | 0.620691489 |  |  |
| Chr21:42866384 | rs1016773134 | 1.1945E-05 | Tolerated | Deleterious | deleterious | 22.4 | 0.722498422 | 0.508220109 |  |  |
| Chr21:42866453 | rs745470783 | 1.1944E-05 | Damaging | Deleterious | deleterious | 23.1 | 0.642889517 | 0.582777899 |  |  |
| Chr21:42866385 | rs749752988 | 1.1943E-05 | Damaging | Deleterious | deleterious | 22.5 | 0.677560398 | 0.714307623 |  |  |
| Chr21:42866393 | rs746532729 | 1.1942E-05 | Tolerated | Neutral | neutral | 7.886 | 0.719605395 | 0.52029868 |  |  |
| Chr21:42861455 | rs781008294 | 1.194E-05 | Tolerated | Neutral | deleterious | 16.9 | 0.721118286 | 0.745959596 |  |  |
| Chr21:42866438 | rs749665029 | 1.1936E-05 | Tolerated | Neutral | neutral | 2.407 | NA | 0.64541829 |  |  |
| Chr21:42851143 | rs1326192818 | 1.1933E-05 | Tolerated | Deleterious | deleterious | 11.82 | 0.611706349 | 0.620614489 |  |  |
| Chr21:42840414 | rs777380293 | 1.193E-05 | Tolerated | Neutral | neutral | 0.046 | 0.534357355 | 0.662867482 |  |  |
| Chr21:42840367 | rs764135262 | 1.193E-05 | Damaging | Deleterious | deleterious | 23.6 | 0.580816925 | 0.621103096 |  |  |
| Chr21:42843810 | rs200744510 | 1.0646E-05 | Tolerated | Neutral | deleterious | 18.58 | 0.65660014 | 0.57604723 |  |  |
| Chr21:42852526 | rs779200981 | 1.0635E-05 | Damaging | Deleterious | deleterious | 26.7 | 0.519061086 | 0.742821977 |  |  |
| Chr21:42851170 | rs763407535 | 1.0609E-05 | Tolerated | Deleterious | deleterious | 5.976 | NA | 0.584077842 |  |  |
| Chr21:42866462 |  | 9.0756E-05 | Damaging | Deleterious | deleterious | 23.7 | 0.520934794 | 0.612844109 |  |  |
| Chr21:42843814 | rs373952557 | 4.8881E-05 | Damaging | Neutral | deleterious | 21.6 | 0.624641902 | 0.600555174 |  |  |
| Chr21:42843738 | rs1343230848 | 4.1872E-05 | Damaging | Deleterious | deleterious | 23.4 | 0.678189851 | 0.615293686 |  |  |
| Chr21:42840430 | rs367866934 | 4.187E-05 | Damaging | Neutral | neutral | 13.97 | 0.414476847 | 0.472650125 |  |  |
| Chr21:42852440 | rs780256128 | 4.1867E-05 | Tolerated | Neutral | neutral | 7.113 | NA | 0.540773001 |  |  |
| Chr21:42851209 | rs141620219 | 2.794E-05 | Tolerated | Neutral | neutral | 25.6 | 0.673238482 | 0.762919372 |  |  |
| Chr21:42866448 | rs1184205003 | 1.3963E-05 | Tolerated | Neutral | neutral | 0.009 | 0.577024217 | 0.875 |  |  |
| Chr21:42843823 |  | 1.3963E-05 | Damaging | Neutral | neutral | 14.89 | 0.554464286 | 0.639154041 |  |  |
| Chr21:42843831 | rs370043174 | 1.3959E-05 | Damaging | Deleterious | deleterious | 21.3 | 0.719235589 | 0.673578923 |  |  |
| Chr21:42852409 | rs1435612851 | 1.3958E-05 | Damaging | Deleterious | deleterious | 24.2 | 0.695344654 | 0.595502499 |  |  |
| Chr21:42842632 | rs893440780 | 1.3958E-05 | Tolerated | Neutral | neutral | 14.87 | 0.519799054 | 0.708798355 |  |  |
| Chr21:42861457 | rs775586470 | 1.3958E-05 | Tolerated | Neutral | neutral | 12.77 | 0.65048841 | 0.610165869 |  |  |
| Chr21:42840381 | rs943194436 | 1.3957E-05 | Damaging | Deleterious | neutral | 15.45 | 0.628481935 | 0.53203125 |  |  |
| Chr21:42860334 |  | 1.3957E-05 | Damaging | Deleterious | deleterious | 23.6 | 0.673299065 | 0.58926467 |  |  |
| Chr21:42840337 |  | 1.3957E-05 | Tolerated | Neutral | neutral | 7.741 | 0.654269924 | 0.58395904 |  |  |
| Chr21:42852413 | rs1287083991 | 1.3956E-05 | Damaging | Neutral | deleterious | 16.79 | 0.605222474 | 0.706348684 |  |  |
| Chr21:42866402 | rs1161378864 | 1.3956E-05 | Tolerated | Neutral | deleterious | 11.58 | 0.619485294 | 0.516356647 |  |  |
| Chr21:42860342 | rs779659161 | 1.3955E-05 | Damaging | Deleterious | deleterious | 24.2 | 0.681930679 | 0.560368274 |  |  |
| Chr21:42842635 | rs1393069401 | 1.3955E-05 | Tolerated | Deleterious | neutral | 5.797 | 0.543893781 | 0.60294124 |  |  |
| Chr21:42845319 | rs778525582 | 1.3954E-05 | Tolerated | Neutral | neutral | 0.001 | 0.640716242 | 0.685625 |  |  |
| Chr21:42845357 | rs1467695759 | 1.3954E-05 | Tolerated | Deleterious | deleterious | 19.26 | 0.707296364 | 0.25 |  |  |
| Chr21:42852427 |  | 1.3954E-05 | Tolerated | Neutral | neutral | 1.424 | 0.676271458 | 0.601894414 |  |  |
| Chr21:42845352 | rs1329369521 | 1.3953E-05 | Damaging | Deleterious | deleterious | 27.5 | 0.823314775 | 0.620252035 |  |  |
