# Supplementary material for: ACE2 and TMPRSS2 SARS-CoV-2 infectivity genes: deep mutational scanning and characterization of missense variants
Source: Hum Mol Genet. 2022 Jul 21;31(24):4183–92. doi: 10.1093/hmg/ddac157 (PMC9759330; doi:10.1093/hmg/ddac157)
Supplement: Supplementary_Table_S3__ddac157 [file supplementary_table_s3__ddac157.docx]

**Supplemental Table S3. Cell numbers collected in each bin for FACS sorting.**

| *ACE2 or TMPRSS2* variants | Bin1 | Bin 2 | Bin3 | Bin 4 |
| --- | --- | --- | --- | --- |
| Experiment 1 | 300,000 | 300,000 | 300,000 | 300,000 |
| Experiment 2 | 300,000 | 300,000 | 300,000 | 300,000 |
| Experiment 3 | 300,000 | 300,000 | 300,000 | 300,000 |
| Experiment 4 | 300,000 | 300,000 | 300,000 | 300,000 |
