# Supplementary material for: ACE2 and TMPRSS2 SARS-CoV-2 infectivity genes: deep mutational scanning and characterization of missense variants
Source: Hum Mol Genet. 2022 Jul 21;31(24):4183–92. doi: 10.1093/hmg/ddac157 (PMC9759330; doi:10.1093/hmg/ddac157)
Supplement: Supplementary_Table_S4_ddac157 [file supplementary_table_s4_ddac157.docx]

| **Supplemental Table S4. Abundance scores and confidence intervals of *ACE2* variants expression from four replicates.** | | | | | | | | | |
| --- | --- | --- | --- | --- | --- | --- | --- | --- | --- |
| variant | e1_score | e2_score | e3_score | e4_score | mean | sd | se | lower_ci | upper_ci |
| c.2158A>G | 0.6675 | 0.684211 | 0.689931 | 0.648129 | 0.672443 | 0.018797 | 0.010852 | 0.654022 | 0.690863 |
| c.77A>G | 0.627883 | 0.576245 | 0.646812 | 0.650442 | 0.625345 | 0.034195 | 0.019743 | 0.591835 | 0.658856 |
| c.2191C>T | 0.527211 | 0.561275 | 0.607784 | 0.690476 | 0.596687 | 0.070712 | 0.040826 | 0.52739 | 0.665983 |
| c.631G>A | 0.706522 | 0.75 | 0.729167 | 0.75 | 0.733922 | 0.02074 | 0.011974 | 0.713598 | 0.754247 |
| c.1402A>G | 0.658745 | 0.63796 | 0.66383 | 0.634401 | 0.648734 | 0.014715 | 0.008496 | 0.634313 | 0.663155 |
| c.2074T>C | 0.708333 | 0.4375 | 0.675439 | 0.575221 | 0.599123 | 0.121716 | 0.070273 | 0.479844 | 0.718403 |
| c.1022A>G | 0.645833 | 0.695946 | 0.724576 | 0.781746 | 0.712025 | 0.05674 | 0.032759 | 0.656421 | 0.76763 |
| c.655C>T | 0.696429 | 0.535714 | NA | 0.722222 | 0.651455 | 0.101061 | 0.058417 | 0.537 | 0.766 |
| c.55T>C | 0.605932 | 0.671875 | 0.710227 | 0.801724 | 0.69744 | 0.081784 | 0.047218 | 0.617293 | 0.777587 |
| c.617A>G | 0.720588 | 0.618421 | 0.659091 | 0.5 | 0.624525 | 0.093035 | 0.053714 | 0.533352 | 0.715698 |
| c.1913A>G | 0.736842 | 0.644737 | 0.815789 | 0.75 | 0.736842 | 0.070449 | 0.040674 | 0.667803 | 0.805881 |
| c.2089A>G | 0.65524 | 0.672414 | 0.736111 | 0.681322 | 0.686272 | 0.034945 | 0.020176 | 0.652026 | 0.720517 |
| c.1640C>G | 0.646376 | 0.626724 | 0.637162 | 0.682213 | 0.648119 | 0.024106 | 0.013917 | 0.624496 | 0.671742 |
| c.1840G>T | 0.6375 | 0.77907 | 0.721154 | 0.701613 | 0.709834 | 0.058371 | 0.033701 | 0.652632 | 0.767037 |
| c.344G>A | 0.75 | 0.37931 | 0.548077 | 0.605263 | 0.570663 | 0.153281 | 0.088497 | 0.42045 | 0.720875 |
| c.1791C>A | 0.775 | 0.708333 | 0.678571 | 0.642857 | 0.70119 | 0.056016 | 0.032341 | 0.646296 | 0.756085 |
| c.2221A>G | 0.633333 | 0.688218 | 0.696429 | 0.722756 | 0.685184 | 0.037576 | 0.021694 | 0.648361 | 0.722008 |
| c.656G>A | 0.704545 | 0.62037 | 0.538462 | 0.597826 | 0.615301 | 0.068799 | 0.039721 | 0.547879 | 0.682723 |
| c.1133A>G | 0.638943 | 0.654857 | 0.670099 | 0.696899 | 0.6652 | 0.024666 | 0.014241 | 0.641027 | 0.689372 |
| c.2147G>A | 0.655682 | 0.65332 | 0.664583 | 0.689914 | 0.665875 | 0.016744 | 0.009667 | 0.649466 | 0.682284 |
| c.22C>T | 0.351544 | 0.354761 | 0.352253 | 0.307108 | 0.341417 | 0.022914 | 0.013229 | 0.318962 | 0.363872 |
| c.1339G>T | 0.5625 | 0.375 | 0.45 | 0.55 | 0.484375 | 0.088609 | 0.051158 | 0.39754 | 0.57121 |
| c.2345C>T | 0.78125 | 0.713636 | 0.607143 | 0.642857 | 0.686222 | 0.077278 | 0.044617 | 0.61049 | 0.761953 |
| c.2315A>G | 0.611257 | 0.64273 | 0.65625 | 0.694129 | 0.651091 | 0.03433 | 0.01982 | 0.617449 | 0.684734 |
| c.787C>T | 0.573864 | 0.578571 | 0.535201 | 0.558333 | 0.561492 | 0.019544 | 0.011284 | 0.542339 | 0.580646 |
| c.1594G>A | 0.65 | 0.512931 | 0.472222 | 0.50641 | 0.535391 | 0.078464 | 0.045301 | 0.458497 | 0.612284 |
| c.1481A>T | 0.695652 | 0.697917 | 0.725 | 0.775 | 0.723392 | 0.036898 | 0.021303 | 0.687233 | 0.759552 |
| c.884A>G | 0.696667 | 0.543919 | 0.552326 | 0.611702 | 0.601153 | 0.07046 | 0.04068 | 0.532103 | 0.670203 |
| c.1449G>C | 0.728774 | 0.752137 | 0.765625 | 0.729167 | 0.743926 | 0.018126 | 0.010465 | 0.726162 | 0.761689 |
| c.1512C>A | 0.767857 | 0.653846 | 0.7125 | 0.527778 | 0.665495 | 0.102939 | 0.059432 | 0.564617 | 0.766373 |
| c.2012G>C | 0.5 | 1 | 0.5 | 1 | 0.75 | 0.288675 | 0.166667 | 0.467104 | 1.032896 |
| c.1709T>C | 0.735294 | 0.644231 | 0.863636 | 0.671875 | 0.728759 | 0.097665 | 0.056387 | 0.633049 | 0.824469 |
| c.2218T>C | 0.727099 | 0.709184 | 0.762821 | 0.939655 | 0.78469 | 0.105689 | 0.061019 | 0.681117 | 0.888263 |
| c.551T>G | 0.657407 | 0.671875 | 0.636364 | 0.66 | 0.656412 | 0.014775 | 0.00853 | 0.641932 | 0.670891 |
| c.513G>C | 0.471264 | 0.663265 | 0.767857 | 0.479651 | 0.595509 | 0.145091 | 0.083769 | 0.453323 | 0.737696 |
| c.1961A>C | 0.759259 | 0.557143 | 0.75 | 0.75 | 0.704101 | 0.098069 | 0.05662 | 0.607995 | 0.800206 |
| c.2328T>A | 0.739362 | 0.567708 | 0.71875 | 0.6 | 0.656455 | 0.085279 | 0.049236 | 0.572884 | 0.740027 |
| c.1258T>C | 0.596354 | 0.615979 | 0.715909 | 0.621302 | 0.637386 | 0.053437 | 0.030852 | 0.585019 | 0.689753 |
| c.1338T>G | 0.630065 | 0.619784 | 0.675305 | 0.650996 | 0.644037 | 0.02456 | 0.01418 | 0.619969 | 0.668105 |
| c.2312A>G | 0.5625 | 0.6 | 0.76087 | 0.564103 | 0.621868 | 0.094271 | 0.054427 | 0.529484 | 0.714252 |
| c.120C>G | 0.625 | 0.708333 | 0.25 | 0.708333 | 0.572917 | 0.218833 | 0.126343 | 0.358465 | 0.787369 |
| c.551T>C | 0.410714 | 0.578125 | 0.775 | 0.7 | 0.61596 | 0.159074 | 0.091841 | 0.46007 | 0.77185 |
| c.2129G>A | 0.654762 | 0.661074 | 0.638235 | 0.657143 | 0.652803 | 0.010055 | 0.005805 | 0.64295 | 0.662657 |
| c.1636A>G | 0.805556 | 0.714286 | 0.5 | 0.55 | 0.64246 | 0.14213 | 0.082059 | 0.503175 | 0.781745 |
| c.109G>A | 0.443548 | 0.546296 | 0.684211 | 0.511905 | 0.54649 | 0.101259 | 0.058462 | 0.447258 | 0.645722 |
| c.2353G>A | 0.70625 | 0.629032 | 0.782051 | 0.648305 | 0.69141 | 0.068763 | 0.0397 | 0.624023 | 0.758796 |
| c.1166C>A | 0.601562 | 0.612245 | 0.573529 | 0.65625 | 0.610897 | 0.034362 | 0.019839 | 0.577223 | 0.644571 |
| c.986A>G | 0.712963 | 0.631443 | 0.62931 | 0.75 | 0.680929 | 0.060306 | 0.034817 | 0.621831 | 0.740028 |
| c.808A>G | 0.671875 | 0.6875 | 0.519231 | 0.6 | 0.619651 | 0.077031 | 0.044474 | 0.544163 | 0.69514 |
| c.2128C>T | 0.625 | 0.58006 | 0.62201 | 0.686813 | 0.628471 | 0.043974 | 0.025389 | 0.585377 | 0.671565 |
| c.1844A>G | 0.637208 | 0.64135 | 0.6415 | 0.63607 | 0.639032 | 0.002803 | 0.001618 | 0.636285 | 0.641779 |
| c.770G>A | 0.672764 | 0.618721 | 0.624016 | 0.602778 | 0.62957 | 0.030178 | 0.017423 | 0.599996 | 0.659144 |
| c.246G>A | 0.654259 | 0.670031 | 0.677045 | 0.698302 | 0.674909 | 0.018276 | 0.010552 | 0.656999 | 0.692819 |
| c.2413T>A | 0.583333 | 0.524194 | 0.621951 | 0.60625 | 0.583932 | 0.042866 | 0.024749 | 0.541924 | 0.62594 |
| c.1399G>A | 0.691176 | 0.717391 | 0.770833 | 0.684211 | 0.715903 | 0.039308 | 0.022695 | 0.677382 | 0.754424 |
| c.1501G>A | 0.669492 | 0.597222 | 0.62766 | 0.614407 | 0.627195 | 0.030828 | 0.017799 | 0.596984 | 0.657406 |
| c.2002G>A | 0.741667 | 0.52707 | 0.609375 | 0.59589 | 0.618501 | 0.089673 | 0.051773 | 0.530622 | 0.706379 |
| c.1163A>T | 0.535392 | 0.680636 | 0.544224 | 0.577922 | 0.584543 | 0.066631 | 0.03847 | 0.519246 | 0.649841 |
| c.517G>A | 0.66875 | 0.714789 | 0.742188 | 0.815789 | 0.735379 | 0.061578 | 0.035552 | 0.675034 | 0.795724 |
| c.2012G>A | 0.792373 | 0.703704 | 0.743421 | 0.71 | 0.737374 | 0.040597 | 0.023439 | 0.69759 | 0.777159 |
| c.2122C>T | 0.651408 | 0.658083 | 0.67975 | 0.678485 | 0.666931 | 0.014342 | 0.00828 | 0.652876 | 0.680986 |
| c.1445G>A | 0.603175 | 0.579918 | 0.54902 | 0.618506 | 0.587655 | 0.03025 | 0.017465 | 0.55801 | 0.617299 |
| c.725C>T | 0.730769 | 0.483333 | 0.482759 | 0.552632 | 0.562373 | 0.116959 | 0.067526 | 0.447756 | 0.67699 |
| c.1825G>A | 0.629801 | 0.644574 | 0.621547 | 0.689366 | 0.646322 | 0.030236 | 0.017456 | 0.616692 | 0.675952 |
| c.872T>A | 0.656389 | 0.665809 | 0.658711 | 0.669511 | 0.662605 | 0.006104 | 0.003524 | 0.656624 | 0.668586 |
| c.596A>G | 0.692308 | 0.625 | 0.576923 | 0.5625 | 0.614183 | 0.058537 | 0.033797 | 0.556817 | 0.671548 |
| c.1013A>G | 0.453704 | 0.738372 | 0.793103 | 0.695652 | 0.670208 | 0.149746 | 0.086456 | 0.52346 | 0.816956 |
| c.2107G>T | 0.729167 | 0.593137 | 0.65 | 0.828947 | 0.700313 | 0.102302 | 0.059064 | 0.600058 | 0.800567 |
| c.994A>T | 0.759901 | 0.641369 | 0.764706 | 0.709302 | 0.71882 | 0.057395 | 0.033137 | 0.662574 | 0.775065 |
| c.1783C>G | 0.503886 | 0.514451 | 0.674688 | 0.513158 | 0.551546 | 0.08223 | 0.047475 | 0.470962 | 0.632129 |
| c.1745G>A | 0.8 | 0.613636 | 0.64881 | 0.660256 | 0.680676 | 0.081986 | 0.047334 | 0.600331 | 0.76102 |
| c.1888G>C | 0.658356 | 0.635935 | 0.676212 | 0.664761 | 0.658816 | 0.016948 | 0.009785 | 0.642207 | 0.675425 |
| c.1125G>T | 0.859375 | 0.694444 | 0.653061 | 0.914894 | 0.780444 | 0.126398 | 0.072976 | 0.656576 | 0.904311 |
| c.578C>A | 0.639706 | 0.632653 | 0.572917 | 0.720588 | 0.641466 | 0.060663 | 0.035024 | 0.582017 | 0.700915 |
| c.2065A>G | 0.711111 | 0.618421 | 0.630682 | 0.717391 | 0.669401 | 0.052093 | 0.030076 | 0.618351 | 0.720451 |
| c.658G>A | 0.680292 | 0.666667 | 0.707071 | 0.671089 | 0.681279 | 0.018107 | 0.010454 | 0.663535 | 0.699024 |
| c.120C>A | 0.568182 | 0.5625 | 0.71875 | 0.638889 | 0.62208 | 0.073218 | 0.042272 | 0.550328 | 0.693832 |
| c.476A>G | 0.697917 | 0.701923 | 0.5 | 0.839286 | 0.684781 | 0.139621 | 0.08061 | 0.547955 | 0.821607 |
| c.103G>A | 0.691558 | 0.525391 | 0.65544 | 0.618812 | 0.6228 | 0.071409 | 0.041228 | 0.552821 | 0.69278 |
| c.2341T>C | 0.5 | 0.8125 | 0.5 | 0.475 | 0.571875 | 0.160849 | 0.092866 | 0.414246 | 0.729504 |
| c.305A>C | 0.611446 | 0.508993 | 0.537313 | 0.612069 | 0.567455 | 0.052447 | 0.03028 | 0.516059 | 0.618852 |
| c.1778C>A | 0.628505 | 0.605634 | 0.635204 | 0.708333 | 0.644419 | 0.04445 | 0.025663 | 0.600858 | 0.68798 |
| c.648C>A | 0.75 | 0.8 | 0.291667 | 0.517857 | 0.589881 | 0.233743 | 0.134952 | 0.360817 | 0.818945 |
| c.1746G>T | 0.651685 | 0.67561 | 0.681102 | 0.691341 | 0.674935 | 0.016815 | 0.009708 | 0.658457 | 0.691413 |
| c.192T>A | 0.694444 | 0.681818 | 0.580357 | 0.72619 | 0.670703 | 0.063057 | 0.036406 | 0.608908 | 0.732497 |
| c.1189A>G | 0.704897 | 0.672619 | 0.715517 | 0.818627 | 0.727915 | 0.063166 | 0.036469 | 0.666013 | 0.789817 |
| c.2302C>T | 0.625 | 0.6 | 0.633333 | 0.572917 | 0.607812 | 0.027236 | 0.015725 | 0.581121 | 0.634504 |
| c.2299G>C | 0.634545 | 0.683857 | 0.728604 | 0.705446 | 0.688113 | 0.040114 | 0.02316 | 0.648801 | 0.727424 |
| c.1510T>A | 0.570313 | 0.651515 | 0.654167 | 0.713115 | 0.647277 | 0.058662 | 0.033868 | 0.58979 | 0.704765 |
| c.907G>A | 0.763158 | 0.807692 | 0.964286 | 0.803571 | 0.834677 | 0.088711 | 0.051218 | 0.747741 | 0.921612 |
| c.899A>G | 0.596774 | 0.634615 | 0.705357 | 0.63 | 0.641687 | 0.045671 | 0.026368 | 0.596929 | 0.686444 |
| c.1063G>A | 0.71 | 0.641304 | 0.69 | 0.633333 | 0.668659 | 0.037241 | 0.021501 | 0.632164 | 0.705155 |
| c.2252G>A | 0.591549 | 0.540541 | 0.735119 | 0.705882 | 0.643273 | 0.092349 | 0.053318 | 0.552773 | 0.733773 |
| c.2176G>C | 0.69084 | 0.625 | 0.714286 | 0.840909 | 0.717759 | 0.090382 | 0.052182 | 0.629186 | 0.806332 |
| c.874G>A | 0.318182 | 0.371429 | 0.483333 | 0.419643 | 0.398147 | 0.070302 | 0.040589 | 0.329252 | 0.467041 |
| c.934G>A | 0.568031 | 0.564841 | 0.672515 | 0.569444 | 0.593708 | 0.052573 | 0.030353 | 0.542187 | 0.645229 |
| c.868A>C | 0.625 | 0.575 | 0.75 | 0.5625 | 0.628125 | 0.08562 | 0.049433 | 0.544219 | 0.712031 |
| c.755A>G | 0.5 | 0.541667 | 0.526224 | 0.446429 | 0.50358 | 0.041803 | 0.024135 | 0.462614 | 0.544546 |
| c.2201C>T | 0.835938 | 0.722561 | 0.77027 | 0.729167 | 0.764484 | 0.052102 | 0.030081 | 0.713425 | 0.815543 |
| c.179A>G | 1 | 0.75 | 0.416667 | 0.833333 | 0.75 | 0.245327 | 0.141639 | 0.509584 | 0.990416 |
| c.172A>C | 0.541667 | 0.9 | 0.85 | 0.71875 | 0.752604 | 0.160054 | 0.092407 | 0.595754 | 0.909455 |
| c.2387A>G | 0.65 | 0.673077 | 0.681818 | 0.535714 | 0.635152 | 0.067637 | 0.03905 | 0.568869 | 0.701436 |
| c.320T>C | 0.617953 | 0.628338 | 0.676724 | 0.6379 | 0.640229 | 0.025658 | 0.014813 | 0.615085 | 0.665373 |
| c.1097T>C | 0.484375 | 0.637931 | 0.632653 | 0.596154 | 0.587778 | 0.071394 | 0.04122 | 0.517813 | 0.657743 |
| c.1718T>C | 0.64527 | 0.663158 | 0.728261 | 0.686111 | 0.6807 | 0.035844 | 0.020694 | 0.645574 | 0.715826 |
| c.2014G>C | 0.5 | 0.555556 | 0.875 | 1 | 0.732639 | 0.243056 | 0.140328 | 0.494449 | 0.970829 |
| c.2086C>A | 0.747619 | 0.731293 | 0.753521 | 0.604027 | 0.709115 | 0.070687 | 0.040811 | 0.639843 | 0.778387 |
| c.275C>T | 0.586957 | 0.681818 | 0.55 | 0.711538 | 0.632578 | 0.076507 | 0.044171 | 0.557603 | 0.707553 |
| c.462C>G | 0.605405 | 0.611364 | 0.656832 | 0.690698 | 0.641075 | 0.040273 | 0.023252 | 0.601608 | 0.680542 |
| c.1880C>T | 0.59 | 0.5 | 0.65 | 0.625 | 0.59125 | 0.065622 | 0.037887 | 0.526942 | 0.655558 |
| c.1279G>T | 0.565217 | 0.557882 | 0.488263 | 0.486364 | 0.524431 | 0.042972 | 0.02481 | 0.48232 | 0.566543 |
| c.202A>G | 0.611111 | 0.691176 | 0.671053 | 0.75 | 0.680835 | 0.057294 | 0.033079 | 0.624688 | 0.736982 |
| c.257A>G | 0.625 | 0.604167 | 0.821429 | 0.75 | 0.700149 | 0.103366 | 0.059679 | 0.598852 | 0.801446 |
| c.61A>G | 0.611111 | 0.5625 | 0.714286 | 0.875 | 0.690724 | 0.138194 | 0.079786 | 0.555297 | 0.826151 |
| c.79A>G | 0.685527 | 0.617991 | 0.681078 | 0.692339 | 0.669234 | 0.034474 | 0.019904 | 0.63545 | 0.703018 |
| c.776T>C | 0.615385 | 0.5 | 0.6875 | 0.625 | 0.606971 | 0.078153 | 0.045122 | 0.530383 | 0.683559 |
| c.2233G>A | 0.629365 | 0.647105 | 0.634717 | 0.660517 | 0.642926 | 0.013883 | 0.008015 | 0.629321 | 0.656531 |
| c.2186C>T | 0.583333 | 0.722222 | 0.8125 | 0.8125 | 0.732639 | 0.108253 | 0.0625 | 0.626553 | 0.838725 |
| c.2411C>T | 0.641304 | 0.617647 | 0.9375 | 0.625 | 0.705363 | 0.155074 | 0.089532 | 0.553394 | 0.857332 |
| c.1055G>T | 0.648148 | 0.622093 | 0.657143 | 0.572917 | 0.625075 | 0.037816 | 0.021833 | 0.588016 | 0.662134 |
| c.977G>A | 0.720588 | 0.596154 | 0.875 | 0.6875 | 0.719811 | 0.116074 | 0.067015 | 0.60606 | 0.833561 |
| c.1540C>G | 0.71875 | 0.75 | 0.454545 | 0.65625 | 0.644886 | 0.132745 | 0.07664 | 0.514799 | 0.774974 |
| c.343C>T | 0.544118 | 0.623016 | 0.652174 | 0.5775 | 0.599202 | 0.047883 | 0.027645 | 0.552277 | 0.646126 |
| c.1677G>C | 0.7 | 0.78125 | 0.9375 | 0.846154 | 0.816226 | 0.100557 | 0.058056 | 0.717682 | 0.91477 |
| c.751G>A | 0.662879 | 0.810185 | 0.689286 | 0.655405 | 0.704439 | 0.07198 | 0.041558 | 0.6339 | 0.774978 |
| c.1343G>A | 0.654412 | 0.570513 | 0.514706 | 0.560345 | 0.574994 | 0.058242 | 0.033626 | 0.517917 | 0.63207 |
| c.1658A>C | 0.687158 | 0.647129 | 0.677536 | 0.660714 | 0.668135 | 0.017763 | 0.010256 | 0.650727 | 0.685542 |
