# Supplementary material for: ACE2 and TMPRSS2 SARS-CoV-2 infectivity genes: deep mutational scanning and characterization of missense variants
Source: Hum Mol Genet. 2022 Jul 21;31(24):4183–92. doi: 10.1093/hmg/ddac157 (PMC9759330; doi:10.1093/hmg/ddac157)
Supplement: Supplementary_Table_S5_ddac157 [file supplementary_table_s5_ddac157.docx]

| **Supplemental Table S5. Abundance scores and confidence intervals of TMPRSS2 isoform1 from four replicates.** | | | | | | | | | |  |
| --- | --- | --- | --- | --- | --- | --- | --- | --- | --- | --- |
| Position | rsID | e1_score | e2_score | e3_score | e4_score | mean | sd | se | lower_ci | upper_ci |
| Chr21:42879909 | rs75603675 | 0.440476 | 0.5625 | 0.444444 | 0.75 | 0.549355 | 0.145249 | 0.08386 | 0.407014 | 0.691697 |
| Chr21:42852497 | rs12329760 | 0.502703 | 0.447545 | 0.391529 | 0.350482 | 0.423065 | 0.066343 | 0.038303 | 0.35805 | 0.48808 |
| Chr21:42866297 | rs61735793 | 0.545455 | 0.589286 | 0.708333 | 0.65 | 0.623268 | 0.071088 | 0.041043 | 0.553604 | 0.692933 |
| Chr21:42866439 | rs61735791 | 0.347222 | 0.333333 | 0.4375 | 0.25 | 0.342014 | 0.076782 | 0.04433 | 0.266768 | 0.417259 |
| Chr21:42866468 | rs61735790 | 0.486842 | 0.689394 | 0.681818 | 0.801724 | 0.664945 | 0.130782 | 0.075507 | 0.536781 | 0.793108 |
| Chr21:42840394 | rs148125094 | 0.857955 | 0.877358 | 0.794643 | 0.770833 | 0.825197 | 0.050606 | 0.029217 | 0.775605 | 0.87479 |
| Chr21:42866301 | rs114363287 | 0.346154 | 0.458333 | 0.833333 | 0.9375 | 0.64383 | 0.285875 | 0.16505 | 0.363678 | 0.923983 |
| Chr21:42861487 | rs147711290 | 0.590909 | 0.3125 | 0.461538 | 0.613636 | 0.494646 | 0.138683 | 0.080068 | 0.35874 | 0.630552 |
| Chr21:42866388 | rs201679623 | 0.286174 | 0.269689 | 0.270394 | 0.264872 | 0.272782 | 0.009259 | 0.005346 | 0.263708 | 0.281855 |
| Chr21:42851157 | rs150554820 | 0.653333 | 0.677536 | 0.621795 | 0.709184 | 0.665462 | 0.037019 | 0.021373 | 0.629184 | 0.70174 |
| Chr21:42845373 | rs61735796 | 0.377717 | 0.473214 | 0.579545 | 0.654762 | 0.52131 | 0.121289 | 0.070026 | 0.402449 | 0.64017 |
| Chr21:42866399 | rs138651919 | 0.450521 | 0.651515 | 0.676056 | 0.639535 | 0.604407 | 0.103711 | 0.059877 | 0.502772 | 0.706041 |
| Chr21:42842623 | rs61735795 | 0.648148 | 0.666667 | 0.678571 | 0.71875 | 0.678034 | 0.029891 | 0.017258 | 0.648741 | 0.707327 |
| Chr21:42845313 | rs142446494 | 0.758621 | 0.677419 | 0.653846 | 0.763889 | 0.713444 | 0.056081 | 0.032378 | 0.658485 | 0.768402 |
| Chr21:42866423 | rs201093031 | 0.616883 | 0.631757 | 0.776042 | 0.693548 | 0.679557 | 0.072383 | 0.04179 | 0.608623 | 0.750492 |
| Chr21:42843882 | rs768173297 | 0.567797 | 0.672414 | 0.709677 | 0.671429 | 0.655329 | 0.06101 | 0.035224 | 0.59554 | 0.715118 |
| Chr21:42843822 | rs565237319 | 0.6 | 0.464286 | 0.75 | 0.75 | 0.641071 | 0.137442 | 0.079352 | 0.506381 | 0.775762 |
| Chr21:42866396 | rs376143876 | 0.469136 | 0.427083 | 0.691667 | 0.632353 | 0.55506 | 0.127013 | 0.073331 | 0.43059 | 0.67953 |
| Chr21:42866301 | rs114363287 | 0.757813 | 0.673913 | 0.744186 | 0.71875 | 0.723665 | 0.036908 | 0.021309 | 0.687496 | 0.759834 |
| Chr21:42845366 | rs150445636 | 0.683824 | 0.666667 | 0.721311 | 0.682584 | 0.688596 | 0.023167 | 0.013375 | 0.665893 | 0.7113 |
| Chr21:42861487 | rs147711290 | 0.502049 | 0.466292 | 0.673077 | 0.74375 | 0.596292 | 0.133443 | 0.077044 | 0.46552 | 0.727064 |
| Chr21:42866328 | rs150389990 | 0.6875 | 0.711538 | 0.607143 | 0.8 | 0.701545 | 0.079377 | 0.045829 | 0.623757 | 0.779334 |
| Chr21:42848536 | rs547544037 | 0.785714 | 0.866667 | 0.733607 | 0.691176 | 0.769291 | 0.075558 | 0.043623 | 0.695246 | 0.843336 |
| Chr21:42879886 | rs1243033377 | 0.772727 | 0.72973 | 0.66129 | 0.693396 | 0.714286 | 0.047954 | 0.027686 | 0.667292 | 0.76128 |
| Chr21:42860333 | rs758778273 | 0.532258 | 0.63 | 0.677083 | 0.62931 | 0.617163 | 0.06086 | 0.035137 | 0.557522 | 0.676804 |
| Chr21:42845360 | rs775494034 | 0.558824 | 0.478261 | 0.608696 | 0.5625 | 0.55207 | 0.054187 | 0.031285 | 0.498968 | 0.605172 |
| Chr21:42839729 | rs1179096287 | 0.615385 | 0.75 | 0.740741 | 0.901515 | 0.75191 | 0.117117 | 0.067618 | 0.637137 | 0.866683 |
| Chr21:42866348 | rs770214639 | 0.59375 | 0.605263 | 0.653846 | 0.690476 | 0.635834 | 0.04478 | 0.025854 | 0.59195 | 0.679718 |
| Chr21:42843873 | rs748528218 | 0.690476 | 0.670068 | 0.660156 | 0.682171 | 0.675718 | 0.013336 | 0.007699 | 0.662649 | 0.688787 |
| Chr21:42852452 | rs748571451 | 0.583333 | 0.640625 | 0.676471 | 0.6875 | 0.646982 | 0.046914 | 0.027086 | 0.601007 | 0.692957 |
| Chr21:42843810 | rs200744510 | 0.663793 | 0.580645 | 0.675926 | 0.69697 | 0.654333 | 0.051002 | 0.029446 | 0.604353 | 0.704314 |
| Chr21:42845367 | rs757466150 | 0.75 | 0.766667 | 0.725 | 0.5875 | 0.707292 | 0.081676 | 0.047156 | 0.62725 | 0.787333 |
| Chr21:42851202 | rs139926880 | 0.614458 | 0.716867 | 0.701923 | 0.719136 | 0.688096 | 0.049682 | 0.028684 | 0.639408 | 0.736784 |
| Chr21:42861476 | rs190265904 | 0.711538 | 0.719697 | 0.717742 | 0.75 | 0.724744 | 0.017193 | 0.009926 | 0.707896 | 0.741593 |
| Chr21:42845259 | rs747772174 | 0.651163 | 0.62963 | 0.725806 | 0.694444 | 0.675261 | 0.04315 | 0.024913 | 0.632975 | 0.717547 |
| Chr21:42848539 | rs944739499 | 0.616667 | 0.563953 | 0.842593 | 0.870968 | 0.723545 | 0.155776 | 0.089937 | 0.570888 | 0.876203 |
| Chr21:42866331 | rs574582815 | 0.305419 | 0.282824 | 0.273973 | 0.265041 | 0.281814 | 0.01733 | 0.010006 | 0.264831 | 0.298797 |
| Chr21:42845396 | rs775404304 | 0.424419 | 0.653409 | 0.583333 | 0.54 | 0.55029 | 0.096047 | 0.055453 | 0.456166 | 0.644414 |
| Chr21:42843887 | rs769164587 | 0.573741 | 0.555233 | 0.635638 | 0.662088 | 0.606675 | 0.050466 | 0.029137 | 0.557219 | 0.656131 |
| Chr21:42843823 | rs775137340 | 0.410714 | 0.25 | 0.5 | 0.5 | 0.415179 | 0.117889 | 0.068063 | 0.29965 | 0.530707 |
| Chr21:42843843 | rs144046631 | 0.573864 | 0.668605 | 0.65625 | 0.816176 | 0.678724 | 0.100824 | 0.058211 | 0.579918 | 0.777529 |
| Chr21:42861463 | rs201949634 | 0.575472 | 0.605 | 0.621212 | 0.533784 | 0.583867 | 0.038384 | 0.022161 | 0.546251 | 0.621483 |
| Chr21:42848512 | rs760039204 | 0.685714 | 0.660494 | 0.765244 | 0.761628 | 0.71827 | 0.05318 | 0.030704 | 0.666154 | 0.770386 |
| Chr21:42866456 | rs150502923 | 0.568182 | 0.55 | 0.666667 | 0.75 | 0.633712 | 0.092935 | 0.053656 | 0.542637 | 0.724787 |
| Chr21:42845397 | rs762854045 | 0.770833 | 0.720588 | 0.722222 | 0.775 | 0.747161 | 0.029796 | 0.017203 | 0.717961 | 0.776361 |
| Chr21:42866283 | rs779085411 | 0.625 | 0.763158 | 0.625 | 0.635417 | 0.662144 | 0.067522 | 0.038984 | 0.595974 | 0.728314 |
| Chr21:42879925 | rs1299493587 | 0.40625 | 0.35 | 0.292169 | 0.284722 | 0.333285 | 0.056722 | 0.032748 | 0.277699 | 0.388872 |
| Chr21:42866495 | rs767591322 | 0.490741 | 0.685484 | 0.638889 | 0.7125 | 0.631903 | 0.098898 | 0.057099 | 0.534985 | 0.728822 |
| Chr21:42879880 | rs916319861 | 0.763158 | 0.65 | 0.558824 | 0.680556 | 0.663134 | 0.084383 | 0.048719 | 0.58044 | 0.745828 |
| Chr21:42879900 | rs1406664235 | 0.657063 | 0.695848 | 0.723684 | 0.726563 | 0.70079 | 0.032274 | 0.018633 | 0.669162 | 0.732417 |
| Chr21:42866505 | rs1466538703 | 0.263636 | 0.279412 | 0.297414 | 0.25463 | 0.273773 | 0.018796 | 0.010852 | 0.255353 | 0.292193 |
| Chr21:42852407 | rs1401630535 | 0.731884 | 0.855072 | 0.815217 | 0.763158 | 0.791333 | 0.054654 | 0.031555 | 0.737773 | 0.844893 |
| Chr21:42866424 | rs1387038867 | 0.666667 | 0.625 | 0.765957 | 0.703125 | 0.690187 | 0.059753 | 0.034498 | 0.631631 | 0.748744 |
| Chr21:42866411 | rs1175715553 | 0.533582 | 0.419118 | 0.625 | 0.45 | 0.506925 | 0.092381 | 0.053336 | 0.416393 | 0.597457 |
| Chr21:42866391 | rs547639377 | 0.5 | 0.25 | 0.6 | 0.5 | 0.4625 | 0.149304 | 0.086201 | 0.316185 | 0.608815 |
| Chr21:42842656 | rs1430677365 | 0.387182 | 0.402778 | 0.482143 | 0.55914 | 0.457811 | 0.079323 | 0.045797 | 0.380075 | 0.535546 |
| Chr21:42839744 | rs1472953828 | 0.696958 | 0.705758 | 0.700399 | 0.713269 | 0.704096 | 0.007107 | 0.004103 | 0.697131 | 0.711061 |
| Chr21:42840325 | rs772900547 | 0.705357 | 0.665663 | 0.757813 | 0.69382 | 0.705663 | 0.038557 | 0.022261 | 0.667878 | 0.743448 |
| Chr21:42866328 | rs150389990 | 0.4375 | 0.465854 | 0.656 | 0.779412 | 0.584691 | 0.162058 | 0.093564 | 0.425877 | 0.743506 |
| Chr21:42839743 | rs936556491 | 0.663886 | 0.709783 | 0.707062 | 0.766026 | 0.711689 | 0.041883 | 0.024181 | 0.670644 | 0.752734 |
| Chr21:42840396 | rs1415259360 | 0.682249 | 0.684645 | 0.669312 | 0.687449 | 0.680914 | 0.008021 | 0.004631 | 0.673053 | 0.688774 |
| Chr21:42861490 | rs1350156643 | 0.605263 | 0.6 | 0.722222 | 0.678571 | 0.651514 | 0.05923 | 0.034196 | 0.59347 | 0.709559 |
| Chr21:42860428 | rs1233717608 | 0.673077 | 0.653846 | 0.8125 | 0.65625 | 0.698918 | 0.076203 | 0.043996 | 0.624241 | 0.773596 |
| Chr21:42839774 | rs1166094493 | 0.389344 | 0.362903 | 0.527778 | 0.625 | 0.476256 | 0.122722 | 0.070853 | 0.355991 | 0.596521 |
| Chr21:42839719 | rs368268847 | 0.75 | 0.694737 | 0.705189 | 0.692661 | 0.710647 | 0.026802 | 0.015474 | 0.684381 | 0.736912 |
| Chr21:42870068 | rs1331618004 | 0.57967 | 0.628173 | 0.581152 | 0.586022 | 0.593754 | 0.023106 | 0.01334 | 0.571111 | 0.616397 |
| Chr21:42843804 | rs1185182900 | 0.625 | 0.833333 | 0.633333 | 0.732143 | 0.705952 | 0.097875 | 0.056508 | 0.610036 | 0.801868 |
| Chr21:42866343 | rs1266959483 | 0.623418 | 0.668919 | 0.682692 | 0.675676 | 0.662676 | 0.02677 | 0.015455 | 0.636442 | 0.68891 |
| Chr21:42860345 | rs1385130606 | 0.69375 | 0.784722 | 0.666667 | 0.759615 | 0.726189 | 0.055193 | 0.031865 | 0.672101 | 0.780276 |
| Chr21:42845268 | rs372563970 | 0.645105 | 0.749027 | 0.824576 | 0.856913 | 0.768905 | 0.094103 | 0.054331 | 0.676686 | 0.861125 |
| Chr21:42845277 | rs776538081 | 0.685897 | 0.671875 | 0.79375 | 0.66 | 0.702881 | 0.061497 | 0.035506 | 0.642614 | 0.763147 |
| Chr21:42845328 | rs140547429 | 0.647059 | 0.685185 | 0.659091 | 0.719697 | 0.677758 | 0.032171 | 0.018574 | 0.646231 | 0.709285 |
| Chr21:42842590 | rs369579311 | 0.628049 | 0.607143 | 0.588235 | 0.556818 | 0.595061 | 0.030239 | 0.017459 | 0.565427 | 0.624695 |
| Chr21:42866376 | rs777667088 | 0.665669 | 0.639916 | 0.64697 | 0.666667 | 0.654805 | 0.013439 | 0.007759 | 0.641636 | 0.667975 |
| Chr21:42866364 | rs753509316 | 0.627358 | 0.646341 | 0.6625 | 0.66129 | 0.649373 | 0.016413 | 0.009476 | 0.633288 | 0.665457 |
| Chr21:42866409 | rs762928261 | 0.555556 | 0.5 | 0.696429 | 0.769231 | 0.630304 | 0.12415 | 0.071678 | 0.508639 | 0.751968 |
| Chr21:42843906 | rs561063944 | 0.703077 | 0.65847 | 0.722222 | 0.716321 | 0.700023 | 0.028835 | 0.016648 | 0.671765 | 0.728281 |
| Chr21:42845376 | rs537370123 | 0.490991 | 0.377193 | 0.675 | 0.6625 | 0.551421 | 0.143315 | 0.082743 | 0.410975 | 0.691867 |
| Chr21:42866360 | rs766068032 | 0.61747 | 0.718354 | 0.697183 | 0.808594 | 0.7104 | 0.078562 | 0.045358 | 0.633411 | 0.78739 |
| Chr21:42843768 | rs745742232 | 0.748366 | 0.773171 | 0.770408 | 0.757317 | 0.762315 | 0.011589 | 0.006691 | 0.750959 | 0.773672 |
| Chr21:42866369 | rs752650649 | 0.421429 | 0.4375 | 0.4 | 0.625 | 0.470982 | 0.103821 | 0.059941 | 0.369239 | 0.572725 |
| Chr21:42839686 | rs541351488 | 0.425926 | 0.575 | 0.659091 | 0.555556 | 0.553893 | 0.09642 | 0.055668 | 0.459403 | 0.648383 |
| Chr21:42860405 | rs199865751 | 0.576087 | 0.555556 | 0.693182 | 0.652174 | 0.61925 | 0.064472 | 0.037223 | 0.556068 | 0.682431 |
| Chr21:42866414 | rs141232947 | 0.67316 | 0.718627 | 0.710843 | 0.74375 | 0.711595 | 0.029219 | 0.016869 | 0.682961 | 0.740229 |
| Chr21:42861500 | rs763645871 | 0.8125 | 0.53125 | 1 | 0.833333 | 0.794271 | 0.19439 | 0.112231 | 0.603772 | 0.98477 |
| Chr21:42845286 | rs530689404 | 0.650538 | 0.653302 | 0.700521 | 0.726351 | 0.682678 | 0.037066 | 0.0214 | 0.646354 | 0.719002 |
| Chr21:42860422 | rs142988104 | 0.545918 | 0.624138 | 0.631068 | 0.66129 | 0.615604 | 0.049178 | 0.028393 | 0.567411 | 0.663797 |
| Chr21:42843832 | rs757214557 | 0.474719 | 0.362745 | 0.6125 | 0.636364 | 0.521582 | 0.127627 | 0.073686 | 0.396509 | 0.646654 |
| Chr21:42866477 | rs774327563 | 0.628788 | 0.658602 | 0.780612 | 0.776923 | 0.711231 | 0.078943 | 0.045578 | 0.633869 | 0.788594 |
| Chr21:42852443 | rs139092674 | 0.461538 | 0.25 | 0.75 | 1 | 0.615385 | 0.32824 | 0.18951 | 0.293715 | 0.937054 |
| Chr21:42840465 | rs762844469 | 0.69754 | 0.686456 | 0.700696 | 0.684609 | 0.692325 | 0.007984 | 0.00461 | 0.684501 | 0.70015 |
| Chr21:42866331 | rs574582815 | 0.615854 | 0.625 | 0.613095 | 0.641892 | 0.62396 | 0.012992 | 0.007501 | 0.611228 | 0.636692 |
| Chr21:42842584 | rs147233451 | 0.49 | 0.542484 | 0.663793 | 0.648649 | 0.586231 | 0.083837 | 0.048403 | 0.504072 | 0.66839 |
| Chr21:42861434 | rs368936645 | 0.595336 | 0.59897 | 0.61085 | 0.616306 | 0.605366 | 0.009853 | 0.005689 | 0.595709 | 0.615022 |
| Chr21:42840423 | rs746555310 | 0.680151 | 0.676302 | 0.688993 | 0.69717 | 0.685654 | 0.009336 | 0.00539 | 0.676505 | 0.694804 |
| Chr21:42879898 | rs765381251 | 0.910714 | 0.638889 | 0.673913 | 0.791667 | 0.753796 | 0.123346 | 0.071214 | 0.632919 | 0.874672 |
| Chr21:42845291 | rs764393597 | 0.619186 | 0.561594 | 0.698113 | 0.702381 | 0.645319 | 0.067666 | 0.039067 | 0.579007 | 0.71163 |
| Chr21:42866336 | rs771209150 | 0.663306 | 0.681402 | 0.701172 | 0.722222 | 0.692026 | 0.025385 | 0.014656 | 0.667149 | 0.716902 |
| Chr21:42866342 | rs377060358 | 0.709064 | 0.68662 | 0.691379 | 0.716783 | 0.700962 | 0.0143 | 0.008256 | 0.686948 | 0.714976 |
| Chr21:42843894 | rs540987630 | 0.528571 | 0.434211 | 0.642857 | 0.75 | 0.58891 | 0.137153 | 0.079185 | 0.454502 | 0.723317 |
| Chr21:42852446 | rs373847134 | 0.59058 | 0.643293 | 0.785714 | 0.789474 | 0.702265 | 0.100864 | 0.058234 | 0.60342 | 0.80111 |
| Chr21:42848513 | rs762108701 | 0.606796 | 0.606796 | 0.606796 | 0.606796 | 0.606796 | 0 | 0 | 0.606796 | 0.606796 |
| Chr21:42852481 | rs759250613 | 0.568182 | 0.6875 | 0.681818 | 0.75 | 0.671875 | 0.075716 | 0.043715 | 0.597675 | 0.746075 |
| Chr21:42860360 | rs760565628 | 0.546875 | 0.647727 | 0.633929 | 0.75 | 0.644633 | 0.083233 | 0.048054 | 0.563066 | 0.726199 |
| Chr21:42851120 | rs148049486 | 0.667431 | 0.664216 | 0.682331 | 0.69186 | 0.67646 | 0.01295 | 0.007477 | 0.663769 | 0.68915 |
| Chr21:42861481 | rs761195761 | 0.857143 | 0.791667 | 0.25 | 0.375 | 0.568452 | 0.30111 | 0.173846 | 0.27337 | 0.863535 |
| Chr21:42842599 | rs755712060 | 0.697183 | 0.773305 | 0.789394 | 0.766556 | 0.75661 | 0.040759 | 0.023532 | 0.716666 | 0.796553 |
| Chr21:42870050 | rs763515247 | 0.875 | 0.55 | 0.625 | 1 | 0.7625 | 0.210654 | 0.121621 | 0.556063 | 0.968937 |
| Chr21:42852453 | rs376158219 | 0.697368 | 0.613481 | 0.42533 | 0.444131 | 0.545078 | 0.132163 | 0.076304 | 0.41556 | 0.674595 |
| Chr21:42852427 | rs751523924 | 0.290644 | 0.267965 | 0.267901 | 0.262804 | 0.272329 | 0.012447 | 0.007186 | 0.260131 | 0.284527 |
| Chr21:42866405 | rs775450506 | 0.4 | 0.5 | 0.6 | 0.7 | 0.55 | 0.129099 | 0.074536 | 0.423485 | 0.676515 |
| Chr21:42839785 | rs773001010 | 0.394231 | 0.375 | 0.653846 | 0.78125 | 0.551082 | 0.199286 | 0.115058 | 0.355785 | 0.746379 |
| Chr21:42845267 | rs146654734 | 0.571429 | 0.8125 | 0.636364 | 0.625 | 0.661323 | 0.104687 | 0.060441 | 0.558732 | 0.763914 |
| Chr21:42845397 | rs762854045 | 0.511792 | 0.47449 | 0.84 | 0.609756 | 0.60901 | 0.164219 | 0.094812 | 0.448078 | 0.769941 |
| Chr21:42845307 | rs757634613 | 0.721154 | 0.685484 | 0.712121 | 0.614583 | 0.683336 | 0.048271 | 0.027869 | 0.636031 | 0.73064 |
| Chr21:42843733 | rs758128660 | 0.488372 | 0.390244 | 0.525 | 0.684783 | 0.5221 | 0.122471 | 0.070709 | 0.40208 | 0.642119 |
| Chr21:42866307 | rs200169208 | 0.763021 | 0.809426 | 0.721854 | 0.748201 | 0.760626 | 0.036719 | 0.0212 | 0.724642 | 0.796609 |
| Chr21:42860437 | rs771653895 | 0.538945 | 0.605691 | 0.7125 | 0.674342 | 0.632869 | 0.076641 | 0.044249 | 0.557763 | 0.707976 |
| Chr21:42843874 | rs772504668 | 0.605586 | 0.605586 | 0.605586 | 0.605586 | 0.605586 | 0 | 0 | 0.605586 | 0.605586 |
| Chr21:42843808 | rs772196502 | 0.608696 | 0.640625 | 0.708333 | 0.75 | 0.676913 | 0.06403 | 0.036968 | 0.614166 | 0.739661 |
| Chr21:42852434 | rs781089181 | 0.426667 | 0.451923 | 0.703704 | 0.764706 | 0.58675 | 0.172387 | 0.099527 | 0.417814 | 0.755686 |
| Chr21:42860412 | rs751035521 | 0.408621 | 0.354247 | 0.570833 | 0.544444 | 0.469536 | 0.104681 | 0.060438 | 0.36695 | 0.572122 |
| Chr21:42852518 | rs766503231 | 0.583333 | 0.583333 | 0.775 | 0.725 | 0.666667 | 0.098366 | 0.056792 | 0.570269 | 0.763064 |
| Chr21:42852504 | rs756213944 | 0.41958 | 0.321138 | 0.541667 | 0.56 | 0.460596 | 0.111929 | 0.064622 | 0.350908 | 0.570285 |
| Chr21:42866381 | rs746151019 | 0.525641 | 0.573529 | 0.654762 | 0.739583 | 0.623379 | 0.094032 | 0.054289 | 0.531229 | 0.715529 |
| Chr21:42866384 | rs1016773134 | 0.703125 | 0.75 | 0.909091 | 0.527778 | 0.722498 | 0.156912 | 0.090593 | 0.568727 | 0.87627 |
| Chr21:42866453 | rs745470783 | 0.666667 | 0.695313 | 0.522959 | 0.68662 | 0.64289 | 0.080848 | 0.046678 | 0.56366 | 0.722119 |
| Chr21:42866385 | rs749752988 | 0.630859 | 0.652778 | 0.704955 | 0.721649 | 0.67756 | 0.042776 | 0.024697 | 0.63564 | 0.719481 |
| Chr21:42866393 | rs746532729 | 0.675824 | 0.746753 | 0.692857 | 0.762987 | 0.719605 | 0.041838 | 0.024155 | 0.678605 | 0.760606 |
| Chr21:42861455 | rs781008294 | 0.736979 | 0.706186 | 0.71 | 0.731308 | 0.721118 | 0.015297 | 0.008832 | 0.706127 | 0.736109 |
| Chr21:42851143 | rs1326192818 | 0.55 | 0.535714 | 0.75 | 0.611111 | 0.611706 | 0.097823 | 0.056478 | 0.515842 | 0.707571 |
| Chr21:42840414 | rs777380293 | 0.442029 | 0.559091 | 0.52381 | 0.6125 | 0.534357 | 0.07154 | 0.041303 | 0.46425 | 0.604465 |
| Chr21:42840367 | rs764135262 | 0.5 | 0.459091 | 0.679245 | 0.684932 | 0.580817 | 0.118148 | 0.068213 | 0.465034 | 0.696599 |
| Chr21:42843810 | rs200744510 | 0.708333 | 0.589286 | 0.696429 | 0.632353 | 0.6566 | 0.055922 | 0.032287 | 0.601798 | 0.711403 |
| Chr21:42852526 | rs779200981 | 0.330769 | 0.365385 | 0.764706 | 0.615385 | 0.519061 | 0.207115 | 0.119578 | 0.316092 | 0.72203 |
| Chr21:42866462 |  | 0.309028 | 0.347222 | 0.772727 | 0.654762 | 0.520935 | 0.22832 | 0.13182 | 0.297186 | 0.744684 |
| Chr21:42843814 | rs373952557 | 0.606061 | 0.529412 | 0.696429 | 0.666667 | 0.624642 | 0.073787 | 0.042601 | 0.552332 | 0.696952 |
| Chr21:42843738 | rs1343230848 | 0.615476 | 0.583333 | 0.738024 | 0.775926 | 0.67819 | 0.093208 | 0.053814 | 0.586848 | 0.769532 |
| Chr21:42840430 | rs367866934 | 0.43125 | 0.355769 | 0.480263 | 0.390625 | 0.414477 | 0.053618 | 0.030956 | 0.361932 | 0.467022 |
| Chr21: 42851209 | rs141620219 | 0.611111 | 0.670732 | 0.661111 | 0.75 | 0.673238 | 0.057462 | 0.033175 | 0.616927 | 0.72955 |
| Chr21:42866448 | rs1184205003 | 0.35274 | 0.392857 | 0.833333 | 0.729167 | 0.577024 | 0.240182 | 0.138669 | 0.34165 | 0.812399 |
| Chr21:42843823 |  | 0.575 | 0.392857 | 0.5 | 0.75 | 0.554464 | 0.150265 | 0.086756 | 0.407207 | 0.701722 |
| Chr21:42843831 | rs370043174 | 0.657895 | 0.883333 | 0.55 | 0.785714 | 0.719236 | 0.145775 | 0.084163 | 0.576379 | 0.862092 |
| Chr21:42852409 | rs1435612851 | 0.565217 | 0.671154 | 0.768692 | 0.776316 | 0.695345 | 0.099086 | 0.057208 | 0.598242 | 0.792448 |
| Chr21:42842632 | rs893440780 | 0.468085 | 0.458333 | 0.541667 | 0.611111 | 0.519799 | 0.071341 | 0.041189 | 0.449887 | 0.589712 |
| Chr21:42861457 | rs775586470 | 0.554688 | 0.668478 | 0.628788 | 0.75 | 0.650488 | 0.081394 | 0.046993 | 0.570724 | 0.730253 |
| Chr21:42840381 | rs943194436 | 0.602564 | 0.581818 | 0.681818 | 0.647727 | 0.628482 | 0.044961 | 0.025958 | 0.584421 | 0.672543 |
| Chr21:42860334 |  | 0.677536 | 0.693452 | 0.626087 | 0.696121 | 0.673299 | 0.032526 | 0.018779 | 0.641424 | 0.705174 |
| Chr21:42840337 |  | 0.618852 | 0.594118 | 0.75 | 0.65411 | 0.65427 | 0.068403 | 0.039493 | 0.587236 | 0.721304 |
| Chr21:42852413 | rs1287083991 | 0.5 | 0.602941 | 0.634615 | 0.683333 | 0.605222 | 0.07755 | 0.044774 | 0.529224 | 0.681221 |
| Chr21:42866402 | rs1161378864 | 0.625 | 0.6875 | 0.602941 | 0.5625 | 0.619485 | 0.052209 | 0.030143 | 0.568321 | 0.67065 |
| Chr21:42860342 | rs779659161 | 0.65 | 0.670455 | 0.776316 | 0.630952 | 0.681931 | 0.064958 | 0.037503 | 0.618273 | 0.745588 |
| Chr21:42842635 | rs1393069401 | 0.461268 | 0.418367 | 0.615385 | 0.680556 | 0.543894 | 0.124327 | 0.07178 | 0.422056 | 0.665732 |
| Chr21:42845319 | rs778525582 | 0.635 | 0.656667 | 0.609694 | 0.661504 | 0.640716 | 0.023676 | 0.013669 | 0.617514 | 0.663918 |
| Chr21:42845357 | rs1467695759 | 0.807692 | 0.646947 | 0.654639 | 0.719907 | 0.707296 | 0.074506 | 0.043016 | 0.634282 | 0.780311 |
| Chr21:42852427 |  | 0.601485 | 0.697761 | 0.678161 | 0.727679 | 0.676271 | 0.053855 | 0.031093 | 0.623495 | 0.729048 |
| Chr21:42845352 | rs1329369521 | 0.816568 | 0.79875 | 0.830169 | 0.847772 | 0.823315 | 0.020769 | 0.011991 | 0.802961 | 0.843668 |
