# Supplementary material for: ACE2 and TMPRSS2 SARS-CoV-2 infectivity genes: deep mutational scanning and characterization of missense variants
Source: Hum Mol Genet. 2022 Jul 21;31(24):4183–92. doi: 10.1093/hmg/ddac157 (PMC9759330; doi:10.1093/hmg/ddac157)
Supplement: Supplementary_Table_S6_ddac157 [file supplementary_table_s6_ddac157.docx]

| **Supplemental Table S6. Abundance scores and confidence intervals of TMPRSS2 isoform 2 from four replicates.** | | | | | | | | | | |
| --- | --- | --- | --- | --- | --- | --- | --- | --- | --- | --- |
| Position | rsID | e1_score | e2_score | e3_score | e4_score | mean | sd | se | lower_ci | upper_ci |
| Chr21:42852497 | rs12329760 | 0.431024 | 0.374356 | 0.446765 | 0.412944 | 0.41627252 | 0.031174 | 0.017998 | 0.385722 | 0.446823 |
| Chr21:42866297 | rs61735793 | 0.333333 | 0.777778 | 0.791667 | 0.75 | 0.66319444 | 0.220589 | 0.127357 | 0.447022 | 0.879367 |
| Chr21:42866439 | rs61735791 | 0.626543 | 0.608911 | 0.598291 | 0.63 | 0.61593618 | 0.014956 | 0.008635 | 0.60128 | 0.630593 |
| Chr21:42866468 | rs61735790 | 0.665816 | 0.64951 | 0.547753 | 0.552941 | 0.60400503 | 0.062352 | 0.035999 | 0.542902 | 0.665108 |
| Chr21:42866301 | rs114363287 | 0.6875 | 0.625 | 0.632979 | 0.691667 | 0.65928635 | 0.035176 | 0.020309 | 0.624814 | 0.693759 |
| Chr21:42861487 | rs147711290 | 0.5 | 0.590909 | 0.71875 | 0.75 | 0.63991477 | 0.115919 | 0.066926 | 0.526317 | 0.753513 |
| Chr21:42866388 | rs201679623 | 0.647059 | 0.626316 | 0.728495 | 0.575758 | 0.6444067 | 0.063555 | 0.036694 | 0.582124 | 0.70669 |
| Chr21:42851157 | rs150554820 | 0.568702 | 0.464539 | 0.495509 | 0.528626 | 0.51434406 | 0.044699 | 0.025807 | 0.47054 | 0.558149 |
| Chr21:42845373 | rs61735796 | 0.592711 | 0.603077 | 0.647857 | 0.61791 | 0.61538888 | 0.023989 | 0.01385 | 0.59188 | 0.638898 |
| Chr21:42866399 | rs138651919 | 0.649718 | 0.688303 | 0.636173 | 0.633956 | 0.65203761 | 0.025161 | 0.014527 | 0.62738 | 0.676695 |
| Chr21:42842623 | rs61735795 | 0.46978 | 0.432292 | 0.460784 | 0.443878 | 0.45168344 | 0.016806 | 0.009703 | 0.435214 | 0.468153 |
| Chr21:42845313 | rs142446494 | 0.625 | 0.642857 | 0.767857 | 0.6875 | 0.68080357 | 0.063711 | 0.036783 | 0.618368 | 0.743239 |
| Chr21:42866423 | rs201093031 | 0.572072 | 0.578571 | 0.582547 | 0.588235 | 0.58035649 | 0.006799 | 0.003926 | 0.573693 | 0.58702 |
| Chr21:42843882 | rs768173297 | 0.75 | 0.514706 | 0.733333 | 0.7 | 0.6745098 | 0.108545 | 0.062668 | 0.568138 | 0.780882 |
| Chr21:42843822 | rs565237319 | 0.602564 | 0.698718 | 0.607843 | 0.457143 | 0.59156701 | 0.099895 | 0.057674 | 0.493672 | 0.689462 |
| Chr21:42866396 | rs376143876 | 0.619277 | 0.66055 | 0.644836 | 0.634956 | 0.6399049 | 0.017326 | 0.010003 | 0.622926 | 0.656884 |
| Chr21:42848546 | rs143597099 | 0.7 | 0.5 | 0.363636 | 0.573529 | 0.53429144 | 0.140592 | 0.081171 | 0.396514 | 0.672069 |
| Chr21:42845366 | rs150445636 | 0.597015 | 0.510638 | 0.578947 | 0.556604 | 0.56080109 | 0.037303 | 0.021537 | 0.524244 | 0.597358 |
| Chr21:42861487 | rs147711290 | 0.606075 | 0.647143 | 0.58589 | 0.613109 | 0.61305406 | 0.025486 | 0.014715 | 0.588078 | 0.63803 |
| Chr21:42848536 | rs547544037 | 0.3125 | 0.45 | 0.291667 | 0.25 | 0.32604167 | 0.086628 | 0.050014 | 0.241148 | 0.410935 |
| Chr21:42860333 | rs758778273 | 0.645833 | 0.625 | 0.5 | 0.666667 | 0.609375 | 0.074875 | 0.043229 | 0.535999 | 0.682751 |
| Chr21:42845360 | rs775494034 | 0.550725 | 0.64759 | 0.65625 | 0.634328 | 0.62222334 | 0.048511 | 0.028008 | 0.574684 | 0.669763 |
| Chr21:42843896 | rs1347220628 | 0.547794 | 0.561224 | 0.60567 | 0.527108 | 0.56044929 | 0.033253 | 0.019199 | 0.527862 | 0.593037 |
| Chr21:42839729 | rs1179096287 | 1 | 1 | 0.8125 | 0.7 | 0.878125 | 0.148034 | 0.085467 | 0.733054 | 1.023196 |
| Chr21:42866348 | rs770214639 | 0.588235 | 0.6 | 0.428571 | 0.5125 | 0.53232668 | 0.079296 | 0.045782 | 0.454618 | 0.610036 |
| Chr21:42843873 | rs748528218 | 0.625 | 0.633333 | 0.656977 | 0.640625 | 0.63898377 | 0.013588 | 0.007845 | 0.625668 | 0.6523 |
| Chr21:42852452 | rs748571451 | 0.733333 | 0.675 | 0.8 | 0.791667 | 0.75 | 0.058135 | 0.033564 | 0.693029 | 0.806971 |
| Chr21:42843810 | rs200744510 | 0.695205 | 0.639831 | 0.665441 | 0.704082 | 0.6761397 | 0.029309 | 0.016922 | 0.647417 | 0.704862 |
| Chr21:42845367 | rs757466150 | 0.75 | 0.6875 | 0.571429 | 0.666667 | 0.66889881 | 0.074002 | 0.042725 | 0.596378 | 0.741419 |
| Chr21:42851202 | rs139926880 | 0.615854 | 0.610169 | 0.579268 | 0.632353 | 0.6094111 | 0.022189 | 0.012811 | 0.587667 | 0.631156 |
| Chr21:42845259 | rs747772174 | 0.644444 | 0.693182 | 0.707143 | 0.656977 | 0.67543647 | 0.02956 | 0.017067 | 0.646468 | 0.704405 |
| Chr21:42866331 | rs574582815 | 0.5 | 0.25 | 0.25 | 0.75 | 0.4375 | 0.239357 | 0.138193 | 0.202935 | 0.672065 |
| Chr21:42845396 | rs775404304 | 0.738095 | 0.675325 | 0.63806 | 0.731481 | 0.69574027 | 0.047663 | 0.027518 | 0.649032 | 0.742449 |
| Chr21:42843887 | rs769164587 | 0.59 | 0.634375 | 0.634804 | 0.634111 | 0.62332243 | 0.022217 | 0.012827 | 0.60155 | 0.645094 |
| Chr21:42843823 | rs775137340 | 0.592308 | 0.545045 | 0.590726 | 0.594792 | 0.58071755 | 0.02384 | 0.013764 | 0.557354 | 0.604081 |
| Chr21:42843843 | rs144046631 | 0.634615 | 0.559524 | 0.65 | 0.734375 | 0.64462855 | 0.071709 | 0.041401 | 0.574355 | 0.714902 |
| Chr21:42866442 | rs554868303 | 0.706522 | 0.75 | 0.604167 | 0.863636 | 0.73108119 | 0.107454 | 0.062039 | 0.625778 | 0.836384 |
| Chr21:42861463 | rs201949634 | 0.643939 | 0.570652 | 0.617925 | 0.580357 | 0.60321831 | 0.033949 | 0.019601 | 0.569949 | 0.636488 |
| Chr21:42848512 | rs760039204 | 0.75 | 0.727273 | 0.5625 | 0.725 | 0.69119318 | 0.086535 | 0.049961 | 0.606391 | 0.775996 |
| Chr21:42866456 | rs150502923 | 0.634167 | 0.632143 | 0.682979 | 0.632692 | 0.64549514 | 0.025004 | 0.014436 | 0.620992 | 0.669998 |
| Chr21:42845397 | rs762854045 | 0.5 | 0.7 | 0.25 | 0.75 | 0.55 | 0.227303 | 0.131233 | 0.327247 | 0.772753 |
| Chr21:42866283 | rs779085411 | 0.625 | 0.45 | 1 | 0.416667 | 0.62291667 | 0.267479 | 0.154429 | 0.360792 | 0.885041 |
| Chr21:42866495 | rs767591322 | 0.636792 | 0.710526 | 0.638462 | 0.662698 | 0.66211968 | 0.034374 | 0.019846 | 0.628434 | 0.695806 |
| Chr21:42866505 | rs1466538703 | 0.620455 | 0.659639 | 0.693396 | 0.637019 | 0.65262714 | 0.03157 | 0.018227 | 0.621689 | 0.683565 |
| Chr21:42852407 | rs1401630535 | 0.464286 | 0.49359 | 0.439655 | 0.471311 | 0.46721053 | 0.022215 | 0.012826 | 0.44544 | 0.488981 |
| Chr21:42866424 | rs1387038867 | 0.583333 | 0.765625 | 0.59375 | 0.583333 | 0.63151042 | 0.089544 | 0.051699 | 0.543758 | 0.719262 |
| Chr21:42866411 | rs1175715553 | 0.553251 | 0.578767 | 0.554825 | 0.60213 | 0.57224328 | 0.023093 | 0.013333 | 0.549612 | 0.594874 |
| Chr21:42842656 | rs1430677365 | 0.719596 | 0.693113 | 0.720574 | 0.712817 | 0.71152507 | 0.01275 | 0.007361 | 0.69903 | 0.72402 |
| Chr21:42839744 | rs1472953828 | 0.626894 | 0.644231 | 0.58156 | 0.607143 | 0.61495696 | 0.026931 | 0.015549 | 0.588565 | 0.641349 |
| Chr21:42840325 | rs772900547 | 0.592105 | 0.691176 | 0.776316 | 0.5 | 0.63989938 | 0.119854 | 0.069198 | 0.522445 | 0.757354 |
| Chr21:42866328 | rs150389990 | 0.632172 | 0.633188 | 0.638889 | 0.601563 | 0.62645282 | 0.016855 | 0.009731 | 0.609935 | 0.64297 |
| Chr21:42839743 | rs936556491 | 0.660819 | 0.653392 | 0.640434 | 0.663804 | 0.65461243 | 0.010417 | 0.006014 | 0.644404 | 0.664821 |
| Chr21:42840396 | rs1415259360 | 0.646552 | 0.525 | 0.821429 | 0.520833 | 0.62845341 | 0.141246 | 0.081549 | 0.490034 | 0.766872 |
| Chr21:42861490 | rs1350156643 | 0.717391 | 0.628049 | 0.738095 | 0.659722 | 0.68581439 | 0.05082 | 0.029341 | 0.636012 | 0.735617 |
| Chr21:42860428 | rs1233717608 | 0.625 | 0.625 | 0.875 | 0.384615 | 0.62740385 | 0.200218 | 0.115596 | 0.431194 | 0.823614 |
| Chr21:42839774 | rs1166094493 | 0.588785 | 0.564754 | 0.606209 | 0.598926 | 0.58966867 | 0.018081 | 0.010439 | 0.571949 | 0.607388 |
| Chr21:42839719 | rs368268847 | 0.593284 | 0.630435 | 0.615079 | 0.597458 | 0.60906384 | 0.017094 | 0.009869 | 0.592312 | 0.625816 |
| Chr21:42843804 | rs1185182900 | 0.454545 | 0.5 | 0.625 | 0.6 | 0.54488636 | 0.080895 | 0.046705 | 0.465611 | 0.624162 |
| Chr21:42866343 | rs1266959483 | 0.677083 | 0.726563 | 0.706522 | 0.673077 | 0.69581112 | 0.02535 | 0.014636 | 0.670968 | 0.720654 |
| Chr21:42860345 | rs1385130606 | 0.705882 | 0.796875 | 0.592105 | 0.710526 | 0.70134723 | 0.083992 | 0.048493 | 0.619036 | 0.783658 |
| Chr21:42845268 | rs372563970 | 0.780347 | 0.74434 | 0.743802 | 0.76466 | 0.75828715 | 0.017622 | 0.010174 | 0.741018 | 0.775556 |
| Chr21:42845277 | rs776538081 | 0.660714 | 0.583333 | 0.556818 | 0.643939 | 0.6112013 | 0.049185 | 0.028397 | 0.563001 | 0.659402 |
| Chr21:42845328 | rs140547429 | 0.715 | 0.701389 | 0.695122 | 0.662791 | 0.69357538 | 0.022137 | 0.012781 | 0.671881 | 0.715269 |
| Chr21:42842590 | rs369579311 | 0.774038 | 0.645 | 0.627778 | 0.744318 | 0.69778361 | 0.072266 | 0.041723 | 0.626964 | 0.768603 |
| Chr21:42866376 | rs777667088 | 0.666667 | 0.65625 | 0.648148 | 0.596774 | 0.64195975 | 0.031063 | 0.017934 | 0.611519 | 0.672401 |
| Chr21:42866364 | rs753509316 | 0.656977 | 0.59375 | 0.75 | 0.664773 | 0.66637487 | 0.064183 | 0.037056 | 0.603477 | 0.729273 |
| Chr21:42866409 | rs762928261 | 0.5625 | 0.65 | 0.916667 | 0.666667 | 0.69895833 | 0.152159 | 0.087849 | 0.549845 | 0.848072 |
| Chr21:42843906 | rs561063944 | 0.610592 | 0.626394 | 0.650621 | 0.611577 | 0.62479606 | 0.018672 | 0.010781 | 0.606497 | 0.643095 |
| Chr21:42845376 | rs537370123 | 0.63 | 0.638372 | 0.643763 | 0.646354 | 0.63962237 | 0.007225 | 0.004172 | 0.632542 | 0.646703 |
| Chr21:42866360 | rs766068032 | 0.618421 | 0.460526 | 0.513889 | 0.657895 | 0.56268275 | 0.091267 | 0.052693 | 0.473243 | 0.652123 |
| Chr21:42843768 | rs745742232 | 0.626923 | 0.631579 | 0.679487 | 0.69403 | 0.65800476 | 0.033782 | 0.019504 | 0.624899 | 0.691111 |
| Chr21:42866369 | rs752650649 | 0.586806 | 0.643519 | 0.609375 | 0.650943 | 0.62266062 | 0.029984 | 0.017311 | 0.593277 | 0.652044 |
| Chr21:42839686 | rs541351488 | 0.640805 | 0.62995 | 0.591667 | 0.567143 | 0.60739115 | 0.03412 | 0.019699 | 0.573954 | 0.640828 |
| Chr21:42860405 | rs199865751 | 0.691176 | 0.637097 | 0.5875 | 0.723684 | 0.65986436 | 0.060023 | 0.034654 | 0.601043 | 0.718686 |
| Chr21:42866414 | rs141232947 | 0.602273 | 0.765957 | 0.685345 | 0.689815 | 0.68584745 | 0.066879 | 0.038613 | 0.620307 | 0.751388 |
| Chr21:42861500 | rs763645871 | 0.678571 | 0.692308 | 0.611111 | 0.513158 | 0.62378703 | 0.081845 | 0.047253 | 0.54358 | 0.703994 |
| Chr21:42845286 | rs530689404 | 0.547872 | 0.453704 | 0.515625 | 0.62069 | 0.53447268 | 0.069502 | 0.040127 | 0.466362 | 0.602584 |
| Chr21:42860422 | rs142988104 | 0.557692 | 0.548077 | 0.56 | 0.56 | 0.55644231 | 0.005682 | 0.003281 | 0.550874 | 0.562011 |
| Chr21:42843832 | rs757214557 | 0.654393 | 0.631868 | 0.59557 | 0.668937 | 0.63769196 | 0.031955 | 0.018449 | 0.606376 | 0.669007 |
| Chr21:42866477 | rs774327563 | 0.5 | 0.3125 | 0.458333 | 0.611111 | 0.47048611 | 0.123496 | 0.0713 | 0.349462 | 0.59151 |
| Chr21:42852443 | rs139092674 | 0.683099 | 0.615385 | 0.683036 | 0.671429 | 0.66323687 | 0.03237 | 0.018689 | 0.631515 | 0.694959 |
| Chr21:42840465 | rs762844469 | 0.605442 | 0.630952 | 0.568627 | 0.602837 | 0.60196472 | 0.02559 | 0.014774 | 0.576887 | 0.627042 |
| Chr21:42866331 | rs574582815 | 0.5 | 0.75 | 0.75 | 0.5 | 0.625 | 0.144338 | 0.083333 | 0.483552 | 0.766448 |
| Chr21:42842584 | rs147233451 | 0.600845 | 0.623081 | 0.598645 | 0.604067 | 0.60665983 | 0.011172 | 0.00645 | 0.595712 | 0.617608 |
| Chr21:42861434 | rs368936645 | 0.610403 | 0.621136 | 0.59944 | 0.607684 | 0.60966589 | 0.008955 | 0.00517 | 0.60089 | 0.618442 |
| Chr21:42840423 | rs746555310 | 0.568182 | 0.646552 | 0.640625 | 0.5 | 0.58883964 | 0.069117 | 0.039905 | 0.521106 | 0.656573 |
| Chr21:42845291 | rs764393597 | 0.633929 | 0.614035 | 0.677305 | 0.653465 | 0.64468349 | 0.027057 | 0.015621 | 0.618168 | 0.671199 |
| Chr21:42866336 | rs771209150 | 0.388889 | 0.75 | 1 | 0.5 | 0.65972222 | 0.27252 | 0.157339 | 0.392658 | 0.926787 |
| Chr21:42866342 | rs377060358 | 0.53125 | 0.611111 | 0.777778 | 0.59375 | 0.62847222 | 0.10528 | 0.060783 | 0.5253 | 0.731644 |
| Chr21:42843894 | rs540987630 | 0.55 | 0.593373 | 0.596831 | 0.582746 | 0.58073774 | 0.02135 | 0.012327 | 0.559815 | 0.601661 |
| Chr21:42852446 | rs373847134 | 0.551306 | 0.546488 | 0.606796 | 0.607798 | 0.57809696 | 0.033777 | 0.019501 | 0.544996 | 0.611198 |
| Chr21:42848513 | rs762108701 | 0.619565 | 0.675595 | 0.705882 | 0.689394 | 0.67260919 | 0.037467 | 0.021632 | 0.635892 | 0.709327 |
| Chr21:42852481 | rs759250613 | 0.5 | 0.375 | 0.458333 | 0.875 | 0.55208333 | 0.221461 | 0.127861 | 0.335055 | 0.769111 |
| Chr21:42860360 | rs760565628 | 0.739362 | 0.643519 | 0.612069 | 0.672619 | 0.66689206 | 0.054273 | 0.031334 | 0.613706 | 0.720078 |
| Chr21:42851120 | rs148049486 | 0.611111 | 0.6 | 0.660714 | 0.706522 | 0.64458678 | 0.049006 | 0.028293 | 0.596562 | 0.692611 |
| Chr21:42842599 | rs755712060 | 0.652985 | 0.670699 | 0.683155 | 0.648907 | 0.66393655 | 0.015926 | 0.009195 | 0.648329 | 0.679544 |
| Chr21:42870050 | rs763515247 | 0.65 | 0.663043 | 0.711538 | 0.863636 | 0.72205458 | 0.098031 | 0.056598 | 0.625986 | 0.818123 |
| Chr21:42852453 | rs376158219 | 0.571429 | 0.6 | 0.711538 | 0.75 | 0.65824176 | 0.086001 | 0.049653 | 0.573962 | 0.742522 |
| Chr21:42852427 | rs751523924 | 0.527778 | 0.442308 | 0.3 | 0.288462 | 0.38963675 | 0.115655 | 0.066774 | 0.276297 | 0.502977 |
| Chr21:42866405 | rs775450506 | 0.563043 | 0.591981 | 0.504167 | 0.546392 | 0.55139576 | 0.03669 | 0.021183 | 0.51544 | 0.587351 |
| Chr21:42866441 | rs770444484 | 0.642857 | 0.703704 | 0.692857 | 0.79375 | 0.708292 | 0.062833 | 0.036277 | 0.646716 | 0.769868 |
| Chr21:42839785 | rs773001010 | 0.599515 | 0.649457 | 0.590909 | 0.626437 | 0.61657924 | 0.026635 | 0.015378 | 0.590478 | 0.642681 |
| Chr21:42845267 | rs146654734 | 0.75 | NA | 0.625 | 0.333333 | 0.56944444 | 0.213817 | 0.151 | 0.327 | 0.811 |
| Chr21:42845397 | rs762854045 | 0.654062 | 0.64986 | 0.641006 | 0.696108 | 0.66025886 | 0.024511 | 0.014151 | 0.636239 | 0.684279 |
| Chr21:42845307 | rs757634613 | 0.607143 | 0.640625 | 0.675 | 0.625 | 0.63694196 | 0.028825 | 0.016642 | 0.608694 | 0.66519 |
| Chr21:42843733 | rs758128660 | 0.60411 | 0.630542 | 0.61662 | 0.628592 | 0.61996588 | 0.012232 | 0.007062 | 0.607979 | 0.631953 |
| Chr21:42866307 | rs200169208 | 0.704545 | 0.522727 | 0.544118 | 0.605263 | 0.59416338 | 0.081476 | 0.04704 | 0.514318 | 0.674009 |
| Chr21:42860437 | rs771653895 | 0.603856 | 0.661192 | 0.605586 | 0.607232 | 0.61946642 | 0.027851 | 0.01608 | 0.592173 | 0.64676 |
| Chr21:42843874 | rs772504668 | 0.582661 | 0.613821 | 0.580128 | 0.613225 | 0.59745882 | 0.01858 | 0.010727 | 0.579251 | 0.615666 |
| Chr21:42843808 | rs772196502 | 0.711111 | 0.711538 | 0.697368 | 0.69186 | 0.70296962 | 0.009908 | 0.00572 | 0.69326 | 0.712679 |
| Chr21:42852434 | rs781089181 | 0.709622 | 0.715863 | 0.754496 | 0.729895 | 0.72746924 | 0.019913 | 0.011497 | 0.707955 | 0.746984 |
| Chr21:42860412 | rs751035521 | 0.632031 | 0.625958 | 0.634851 | 0.624293 | 0.62928337 | 0.004983 | 0.002877 | 0.6244 | 0.634167 |
| Chr21:42852518 | rs766503231 | 0.5 | 0.642857 | 0.416667 | 0.575 | 0.53363095 | 0.097388 | 0.056227 | 0.438192 | 0.62907 |
| Chr21:42852504 | rs756213944 | 0.602873 | 0.615512 | 0.621743 | 0.629352 | 0.61737006 | 0.0112 | 0.006466 | 0.606395 | 0.628345 |
| Chr21:42866381 | rs746151019 | 0.56 | 0.605932 | 0.618421 | 0.698413 | 0.62069149 | 0.057582 | 0.033245 | 0.564262 | 0.677121 |
| Chr21:42866384 | rs1016773134 | 0.48913 | 0.575 | 0.5 | 0.46875 | 0.50822011 | 0.046366 | 0.026769 | 0.462782 | 0.553658 |
| Chr21:42866453 | rs745470783 | 0.628788 | 0.583333 | 0.634615 | 0.484375 | 0.5827779 | 0.069492 | 0.040121 | 0.514677 | 0.650879 |
| Chr21:42866385 | rs749752988 | 0.663934 | 0.703488 | 0.754808 | 0.735 | 0.71430762 | 0.039678 | 0.022908 | 0.675424 | 0.753191 |
| Chr21:42866393 | rs746532729 | 0.586207 | 0.445313 | 0.492857 | 0.556818 | 0.52029868 | 0.063386 | 0.036596 | 0.458181 | 0.582416 |
| Chr21:42861455 | rs781008294 | 0.85 | 0.772727 | 0.75 | 0.611111 | 0.7459596 | 0.099568 | 0.057486 | 0.648385 | 0.843534 |
| Chr21:42866438 | rs749665029 | 0.621429 | 0.616279 | 0.568966 | 0.775 | 0.64541829 | 0.089556 | 0.051705 | 0.557655 | 0.733182 |
| Chr21:42851143 | rs1326192818 | 0.596774 | 0.663462 | 0.638889 | 0.583333 | 0.62061449 | 0.037095 | 0.021417 | 0.584262 | 0.656967 |
| Chr21:42840414 | rs777380293 | 0.673611 | 0.671233 | 0.637218 | 0.669408 | 0.66286748 | 0.017186 | 0.009922 | 0.646026 | 0.679709 |
| Chr21:42840367 | rs764135262 | 0.632251 | 0.62664 | 0.61086 | 0.614662 | 0.6211031 | 0.010022 | 0.005786 | 0.611281 | 0.630925 |
| Chr21:42843810 | rs200744510 | 0.5625 | 0.608974 | 0.522059 | 0.610656 | 0.57604723 | 0.042349 | 0.02445 | 0.534546 | 0.617548 |
| Chr21:42852526 | rs779200981 | 0.759336 | 0.710145 | 0.754707 | 0.7471 | 0.74282198 | 0.022361 | 0.01291 | 0.720908 | 0.764735 |
| Chr21:42851170 | rs763407535 | 0.57 | 0.618056 | 0.587209 | 0.561047 | 0.58407784 | 0.025119 | 0.014503 | 0.559462 | 0.608694 |
| Chr21:42866462 | | 0.609171 | 0.621716 | 0.603539 | 0.61695 | 0.61284411 | 0.008075 | 0.004662 | 0.604931 | 0.620758 |
| Chr21:42843814 | rs373952557 | 0.576923 | 0.55 | 0.65625 | 0.619048 | 0.60055517 | 0.046755 | 0.026994 | 0.554736 | 0.646375 |
| Chr21:42843738 | rs1343230848 | 0.576484 | 0.62931 | 0.637113 | 0.618267 | 0.61529369 | 0.027004 | 0.015591 | 0.588831 | 0.641757 |
| Chr21:42840430 | rs367866934 | 0.462121 | 0.45614 | 0.482143 | 0.490196 | 0.47265013 | 0.016139 | 0.009318 | 0.456834 | 0.488466 |
| Chr21:42852440 | rs780256128 | 0.535714 | 0.5 | 0.619565 | 0.507813 | 0.540773 | 0.054719 | 0.031592 | 0.487149 | 0.594397 |
| Chr21:42851209 | rs141620219 | 0.8125 | 0.803571 | 0.727273 | 0.708333 | 0.76291937 | 0.052793 | 0.03048 | 0.711184 | 0.814655 |
| Chr21:42866448 | rs1184205003 | 1 | NA | 0.875 | 0.75 | 0.875 | 0.125 | 0.0884 | 0.734 | 1.02 |
| Chr21:42843823 | | 0.644781 | 0.631229 | 0.63434 | 0.646266 | 0.63915404 | 0.007488 | 0.004323 | 0.631816 | 0.646492 |
| Chr21:42843831 | rs370043174 | 0.698052 | 0.665663 | 0.666667 | 0.663934 | 0.67357892 | 0.016354 | 0.009442 | 0.657552 | 0.689606 |
| Chr21:42852409 | rs1435612851 | 0.681818 | 0.586957 | 0.588235 | 0.525 | 0.5955025 | 0.064671 | 0.037338 | 0.532127 | 0.658878 |
| Chr21:42842632 | rs893440780 | 0.698347 | 0.702652 | 0.72306 | 0.711134 | 0.70879836 | 0.010891 | 0.006288 | 0.698125 | 0.719472 |
| Chr21:42861457 | rs775586470 | 0.666667 | 0.561321 | 0.6 | 0.612676 | 0.61016587 | 0.043542 | 0.025139 | 0.567495 | 0.652837 |
| Chr21:42840381 | rs943194436 | 0.5375 | 0.575 | 0.5 | 0.515625 | 0.53203125 | 0.032514 | 0.018772 | 0.500169 | 0.563894 |
| Chr21:42860334 | | 0.625 | 0.62037 | 0.561688 | 0.55 | 0.58926467 | 0.03893 | 0.022477 | 0.551113 | 0.627416 |
| Chr21:42840337 | | 0.617647 | 0.56875 | 0.546875 | 0.602564 | 0.58395904 | 0.032081 | 0.018522 | 0.55252 | 0.615398 |
| Chr21:42852413 | rs1287083991 | 0.75 | 0.657895 | 0.73 | 0.6875 | 0.70634868 | 0.041505 | 0.023963 | 0.665675 | 0.747022 |
| Chr21:42866402 | rs1161378864 | 0.542857 | 0.578125 | 0.527778 | 0.416667 | 0.51635665 | 0.069728 | 0.040258 | 0.448024 | 0.584689 |
| Chr21:42860342 | rs779659161 | 0.485294 | 0.6 | 0.628906 | 0.527273 | 0.56036827 | 0.065827 | 0.038005 | 0.495859 | 0.624878 |
| Chr21:42842635 | rs1393069401 | 0.586614 | 0.632407 | 0.594318 | 0.598425 | 0.60294124 | 0.020245 | 0.011688 | 0.583102 | 0.622781 |
| Chr21:42845319 | rs778525582 | 0.73 | 0.65 | 0.6875 | 0.675 | 0.685625 | 0.03344 | 0.019307 | 0.652854 | 0.718396 |
| Chr21:42845357 | rs1467695759 | 0.25 | NA | 0.25 | 0.25 | 0.25 | 0 | 0 | 0.25 | 0.25 |
| Chr21:42852427 | | 0.640086 | 0.626404 | 0.576087 | 0.565 | 0.60189441 | 0.036908 | 0.021309 | 0.565725 | 0.638063 |
| Chr21:42845352 | rs1329369521 | 0.598214 | 0.628378 | 0.577586 | 0.676829 | 0.62025204 | 0.043101 | 0.024884 | 0.578014 | 0.66249 |
